# Regional differences in health, diet and weaning patterns amongst the first Neolithic farmers of central Europe

**DOI:** 10.1038/srep29458

**Published:** 2016-07-07

**Authors:** Abigail Ash, Michael Francken, Ildikó Pap, Zdeněk Tvrdý, Joachim Wahl, Ron Pinhasi

**Affiliations:** 1 School of Archaeology, University College Dublin, Dublin 4, Republic of Ireland; 2 Institute for Archaeological Sciences and Senckenberg Center for Human Evolution and Paleoenvironment, University of Tübingen, Germany; 3 Department of Anthropology, Hungarian Natural History Museum, Budapest, Hungary; 4 Anthropos Institute, Moravian Museum, Zelný trh 6, Brno, Czech Republic; 5 State Office for Cultural Heritage Management Baden-Württemberg, Osteology, D-78467 Konstanz, Germany; 6 Institute for Archaeological Sciences, WG Palaeoanthropology, University of Tübingen, Germany; 7 Earth Institute, University College Dublin, Dublin 4, Republic of Ireland

## Abstract

Across much of central Europe, the *Linearbandkeramik* (LBK) represents the first Neolithic communities. Arising in Transdanubia around 5500 cal. BC the LBK spread west to the Rhine within two to three hundred years, carrying elements of a mixed agricultural economy and a relatively homogeneous material culture. Colonisation of new regions during this progress would have required economic adaptations to varied ecological conditions within the landscape. This paper investigates whether such adaptation at a local scale affected health patterns and altered the dietary habits of populations that otherwise shared a common cultural and biological origin. Analysis of non-specific stress (linear enamel hypoplasia, porotic hyperostosis, cribra orbitalia) within five LBK populations from across central Europe in conjunction with published carbon and nitrogen stable isotope data from each site revealed a high prevalence of porotic hyperostosis and cribra orbitalia in western populations that was associated with a lower animal protein intake. Hypoplastic enamel was more frequently observed in eastern populations however, and may reflect geographic differences in childhood morbidity and mortality as a result of variation in social practices relating to weaning. Local socio-economic adaptations within the LBK were therefore an important factor in the exposure of populations to non-specific stress.

Settlements of the *Linearbandkeramik* (LBK) culture, identified by their characteristic longhouse architecture and linearly incised pottery, are found across much of central Europe during the late sixth and early fifth millennia BC. The culture first arose in Transdanubia around 5500 cal. BC^1^,^2^, practicing a small-scale intensive garden agriculture based on the cultivation of five staple crops (einkorn and emmer wheat, barley, peas, and lentils) and the husbandry of four animals (cattle, sheep, goats, and pigs^3^,^4^). Rapid spread of the LBK from Transdanubia to the Rhine by 5300 cal. BC suggests a rapid diffusion of the culture and recent studies of genomic data from 17 LBK specimens from Eastern Hungary^5^, Transdanubia, and Germany^6^,^7^ indicate a close genetic affinity between LBK populations across this distribution.

Carbon and nitrogen stable isotope analysis of bones and teeth from LBK populations suggests that they were consuming a mixed terrestrial diet and that this differed little depending on the age, sex, or social status of individuals^8^,^9^,^10^. Small differences in the proportions of proteins and carbohydrates consumed by males and females may, however, have existed at some sites^11^ and dietary differences across the geographic landscape may be identified in the archaeological record^12^.

Analysis of palaeobotanical data and faunal remains from a number of sites has given an insight into to some of the spectrum of foods consumed as part of the LBK diet, and how this diet apparently varied by region. Barley grains are frequently found within settlements from the Carpathian Basin and the Neckar region of Germany, but not over the landscape in between^13^. Greater proportions of einkorn than emmer wheat and the presence of the opium poppy further suggest slightly different subsistence at western German LBK sites^3^,^4^. Exploitation of wild animals and the inclusion of flint or bone arrowheads in graves are more frequent at the western limits of the LBK, possibly indicating a greater emphasis on hunting in this area^14^,^15^. Differences in the manufacture and decoration of fineware pottery, orientation and structuring of longhouses, and burial in cemeteries are observed along an east to west trajectory of the LBK archaeological record^15^,^16^,^17^. The archaeological data suggests that small variations in environmental and ecological conditions across central Europe may have forced local adaptations in diet and other behaviours as LBK populations expanded.

Little palaeopathological data are available for the Neolithic of central Europe (Supplementary Table S1) so it is not known how variations in diet and behaviour may have affected the health of LBK individuals. Non-specific stress, experienced during the lifetime of an individual, is an indicator of periods during which normal growth and repair processes are disrupted. Conditions affecting the skeletal system, such as porotic hyperostosis, cribra orbitalia, and linear enamel hypoplasia, may be suggestive of periods of nutritional or immunological stress suffered by individuals and may be observed at high frequency in prehistoric populations^18^,^19^,^20^. Porotic hyperostosis and cribra orbitalia affect the cranial vault and orbital roof, respectively, and may be identified as porosity of the outer table of bone resulting from expansion of the inner spongy bone^20^, while linear enamel hypoplasia are bands of decreased enamel thickness observed on tooth crowns resulting from the disruption of normal enamel formation during childhood^18^.

This study investigated differences in the experience of such biological stress between western, central and eastern regions of the LBK (Fig. 1) through comparison of the prevalence of porotic hyperostosis, cribra orbitalia, and linear enamel hypoplasia, and also of the age at formation of hypoplastic enamel defects. Analysis was conducted on a total of 511 skeletons from five Early Neolithic collections: Schwetzingen, Stuttgart-Mühlhausen, Vedrovice, Nitra-Horné Krškany, and Polgár-Ferenci-hát (Table 1, see also Supplementary Tables S2 and S3). Both inter-regional variations in the frequencies of these indicators in the overall populations, and intra-cemetery variations in stress within the adult male, adult female, and juvenile subsets of populations were examined. Comparison of results with dietary profiles constructed from published light stable isotope data for each population then facilitated discussion of stress as a response to variable access to dietary resources across the geographic distribution of the LBK.

## Results

### Prevalence of non-specific stress

True prevalence of all non-specific indicators of stress and results of chi-square comparisons between demographic groupings within each population are shown in Table 2. Table 3 further highlights variation in the prevalence of indicators between populations. Little inter-population variability was witnessed among dental defects. Prevalence of linear enamel hypoplasia did not differ significantly across the populations studied, ranging from 11.58% at Vedrovice to 16.81% at Nitra-Horné Krškany. In contrast, significant inter-population differences existed in the prevalence of porotic hyperostosis and cribra orbitalia (PH: χ^2^ = 73.91, df = 4, p < 0.001; CO: χ^2^ = 43.42, df = 2, p < 0.001, Fig. 2, Table 3). The highest prevalence of porotic hyperostosis was found at Stuttgart-Mühlhausen (80.23%), while Schwetzingen had the highest prevalence of cribra orbitalia (53.97%). Both of these populations were located within the western LBK region. When grouped into regions, differences in the prevalence of linear enamel hypoplasia remained non-significant but the prevalence of both porotic hyperostosis and cribra orbitalia was highest in the west (65.06% PH, 50.81% CO) followed by eastern (47.30% PH, 25.00% CO) and then central populations (38.16% PH, 23.73% CO). These inter-regional differences in porotic hyperostosis and cribra orbitalia were also statistically significant (PH: χ^2^ = 14.97, df = 2, p = 0.001; CO: χ^2^ = 21.06, df = 2, p < 0.001).

The true prevalence of porotic hyperostosis was greater in males than females in all populations except Stuttgart-Mühlhausen, where male and female prevalence was equal (87.50% for both), but differences in prevalence were only statistically significant for Vedrovice (p = 0.033) and Polgár-Ferenci-hát (p < 0.001). Prevalence of cribra orbitalia differed significantly between males and females at every site but Vedrovice. Males more frequently displayed orbital lesions at Stuttgart-Mühlhausen and Schwetzingen, while female prevalence was higher at Nitra-Horné Krškany and Polgár-Ferenci-hát (Table 2). No significant difference was found at any site between the male and female prevalence of linear enamel hypoplasia, nor were significant differences seen in the prevalence of males and females affected between sites (p = 0.376 and p = 0.612, respectively). Prevalence of males and females affected by porotic hyperostosis and cribra orbitalia did differ significantly across the LBK (Table 3). Both males and females showed a high level of porotic hyperostosis at Stuttgart-Mühlhausen (87.50% for both), while cribra orbitalia was highest amongst males at Schwetzingen (53.33%) and females from Nitra-Horné Krškany (46.15%). This was further evident when populations were grouped by region. Prevalence of porotic hyperostosis and cribra orbitalia amongst males varied significantly between geographic regions of the LBK (PH: χ^2^ = 21.46, df = 2, p < 0.001; CO: χ^2^ = 60.87, df = 2, p < 0.001), but amongst females only the level of porotic hyperostosis differed significantly (PH: χ^2^ = 28.31, df = 2, p < 0.001; CO: χ^2^ = 5.08, df = 2, p = 0.079) and neither males nor females were particularly variable in the expression of linear enamel hypoplasia.

Porotic hyperostosis was always found at higher prevalence amongst adults than juveniles and this result was statistically significant at all sites apart from Vedrovice where the prevalence amongst both adults and juveniles was low. Conversely juveniles displayed a higher prevalence of cribra orbitalia at every site except Nitra-Horné Krškany (Table 3). Adult and juvenile prevalence of linear enamel hypoplasia differed significantly only within Nitra-Horné Krškany and Polgár-Ferenci-hát, where juvenile prevalence was particularly high (50.00% at Nitra-Horné Krškany and 34.72% at Polgár-Ferenci-hát). As a result of this, juveniles were the only demographic grouping to show significant inter-site and inter-region variability in the prevalence of linear enamel hypoplasia (inter-site: χ^2^ = 69.74, df = 4, p < 0.001; inter-region: χ^2^ = 9.63, df = 4, p = 0.008). Both adult and juvenile prevalence of porotic hyperostosis and cribra orbitalia also varied significantly between populations and between regions (Table 3).

### Age at onset of non-specific stress

Table 4 shows the average age at onset of linear enamel hypoplasia formation within all five populations, this ranged from an early 0.7 years in individual HK23 from Nitra-Horné Krškany, to a late 6.2 years in individual SW109(96) from Schwetzingen (See Supplementary Tables S4 and S5 for results of the Kruskal-Wallis analysis of variance in age at onset). Both of these individuals were juveniles. Variation between sites was significant (H = 31.90, df = 4, p < 0.001) with a difference of almost one year between the population with the earliest average onset (Nitra-Horné Krškany: 2.9 years) and the population with the latest (Schwetzingen and Stuttgart-Mühlhausen: 3.9 years). Adult males and juveniles showed variation in onset (Fig. 3). Males differed significantly in onset between Schwetzingen and Polgár-Ferenci-hát (p = 0.012) while juveniles displayed a much greater degree of variation. Juvenile individuals at Schwetzingen and Stuttgart-Mühlhausen had a significantly later average age at onset of linear enamel hypoplasia formation than cohorts from Nitra-Horné Krškany and Polgár-Ferenci-hát.

Average age at onset in the dentition of females was 0.75 years later than the average age at onset in the dentition of males at Schwetzingen (p = 0.006). No other population evidenced a significant difference between males and females. Onset in teeth from individuals dying during immaturity was significantly earlier on average than onset in the teeth of those individuals surviving to adulthood within the three populations from the central and eastern LBK (Supplementary Table S5), while no difference was observed in the dentition from the western LBK populations.

### Stable isotopes

Variation in carbon and nitrogen isotopic ratios between the five LBK populations was statistically significant (Kruskal-Wallis: δ^13^C - H = 82.87, df = 3, p < 0.001; δ^15^N - H = 169.08, df = 3, p < 0.001). Vedrovice and Schwetzingen displayed lower δ^15^N values than Nitra-Horné Krškany and Polgár-Ferenci-hát, and individuals from Stuttgart-Mühlhausen, Schwetzingen and Nitra-Horné Krškany had more negative δ^13^C than Vedrovice and Polgár-Ferenci-hát (Fig. 4). Application of a post-hoc pairwise Wilcoxon rank sum test indicated that all population pairings differed significantly except Nitra-Horné Krškany/Schwetzingen and Vedrovice/Polgár-Ferenci-hát for δ^13^C, and Nitra-Horné Krškany/Polgár-Ferenci-hát for δ^15^N. At Vedrovice δ^15^N of adults was 0.33‰ higher than that of juveniles and δ^15^N of adult males was 0.44‰ higher than females. Both of these differences were small but significant (two-group Mann-Whitney U test, p = 0.029 and p < 0.001, respectively). Male-female and adult-juvenile isotope ratios did not differ significantly at any other site.

## Discussion

Experience of stress within the LBK appears to have differed significantly from east to west. Porotic hyperostosis and cribra orbitalia were both found at high prevalence in the western LBK skeletal collections of Schwetzingen and Stuttgart-Mühlhausen, suggesting greater nutritional or immunological stress within farming populations towards the western limits of this archaeological culture^20^. Prevalence of linear enamel hypoplasia was more uniform across the LBK, although prevalence amongst juveniles was particularly high in the more easterly populations of Nitra-Horné Krškany and Polgár-Ferenci-hát where average age at onset of linear enamel hypoplasia formation was also significantly younger for juveniles than adults. Findings from this study correspond with palaeopathological data previously published on LBK populations, which also hint at a geographic gradient in skeletal indicators of stress (Supplementary Table S1). High prevalence of cribra orbitalia at the sites of Rutzing (55.60%) and Schletz (55.22%)^21^ in Austria is similar to the prevalence here observed at both Schwetzingen (53.97%) and Stuttgart-Mühlhausen (47.54%). Further east, more moderate prevalence of this pathological condition reported from the site of Füzesabony-Gubakút (12.20%)^22^, Hungary, also corresponds with lower relative levels of the condition in eastern LBK populations. Although broad continental-scale patterns in the health of Early Neolithic populations are difficult to make on the basis of this analysis of just five sites, these results would nevertheless suggest that predictable differences may exist from east to west and that greater investigation of palaeopathology within the LBK is needed to test this hypothesis.

Lower δ^15^N values at Schwetzingen and Vedrovice than Nitra-Horné Krškany or Polgár-Ferenci-hát may suggest a lower intake of animal proteins or a lack of manure-fertilised produce in the diet of these populations^23^. Some variation in isotopic levels of dietary carbon may be expected as a result of environmental differences across the landscape of central Europe^12^, although significantly more negative δ^13^C values at Nitra-Horné Krškany, Schwetzingen and Stuttgart-Mühlhausen could further indicate consumption of a greater proportion of food from closed canopy environments^24^. Domesticates may have been browsing woodland vegetation in these locations, but it is also possible that western isotope values may reflect food obtained from wild resources, as indicated by the high frequency of wild fauna and arrowheads in the western LBK archaeological record^14^,^15^. Wild fauna accounts for just 5–10% of animal remains recovered from LBK settlements in eastern areas^25^ but up to 39% of assemblages from the Baden-Württemberg area of southern Germany^14^. The hunting of wild fauna remained a component of agricultural subsistence into the Iron Age and beyond^26^ but the increased emphasis on this activity in the western LBK perhaps indicates a greater economic need for hunted meat and may suggest that early agriculture was not as successful in western climates as it appears to have been in eastern Europe and the Balkans. Further reliance on barley, einkorn wheat and poppy seeds, which are more tolerant of fluctuating climatic and soil conditions than other Neolithic staples^3^,^4^, may indicate areas of poor agricultural productivity at the western limits of the LBK^27^.

If conditions were less optimal for Neolithic domesticates in the western LBK, the risk of crop failure and food deficit may have been greater and may have led to a greater reliance on crops that would grow more predictably and an increased utilisation of wild resources to supplement the diet. Low δ^15^N values from Schwetzingen arguably indicate that consumption of meat may have been significantly reduced compared to other populations, while enriched nitrogen values of fauna from Heilbronn may suggest intensive manuring of crops in an attempt to increase yield at other sites^23^. Evidence for the diversification of subsistence strategies and retention of productive land within family lineages in the western LBK^28^,^29^ could also represent responses to lower agricultural productivity.

High rates of nutritional stress and disease are associated with periods of deficit in modern populations from low-income countries and may also be witnessed in the skeletons of historic famine victims^30^,^31^,^32^,^33^. Moderate levels of nutritional stress across the LBK coupled with lower agricultural productivity, and hence increased nutritional stress, in western populations would be consistent with the geographic patterning of non-specific indicators of stress highlighted in the present study. Differences in the experience of stress between populations would not be entirely unexpected as each population is subject to unique ecological conditions, however the clear distinction in prevalence of porotic hyperostosis and cribra orbitalia between more easterly and more westerly located sites lends this pattern significance despite the small sample size. Populations from these sites share a high degree of genetic affinity^5^,^6^,^7^ and are broadly comparable in their material culture, although regional variation along an east-west axis^15^,^16^,^17^ coincides with patterns in biological stress observed here and may reflect larger differences in subsistence practices developing as a response to economic need.

East-west patterning in male-female and adult-juvenile prevalence of non-specific stress could further reflect behavioural differences that echo the variation in material culture observed across the LBK distribution. Cribra orbitalia was more prevalent in males within western LBK populations but higher in females from Nitra-Horné Krškany and Polgár-Ferenci-hát. Assessment of tibial cross-sectional geometry within a time-series of central European populations^34^ showed that biomechanical values were particularly high at Schwetzingen and Stuttgart-Mühlhausen when compared to populations from other regions of the LBK and later time periods. This was suggestive of increased mobility, particularly across rough terrain, and could relate to a diversification of subsistence strategies in the western LBK and the greater exploitation of upland resources^29^, which may have affected males more than females although why such a pattern would occur is uncertain. High levels of activity increase the metabolic demand for iron and may be associated with an increased risk of anemia in modern athletes^35^. Similar activity in western LBK males may have increased the risk of iron-deficiency and development of cribra orbitalia beyond the level experienced in females from iron loss during pregnancy and menstruation^36^.

Higher juvenile prevalence of cribra orbitalia and linear enamel hypoplasia, particularly in eastern populations, where significant differences were also observed in the average age at onset of enamel hypoplasia formation between adults and juveniles, may signal greater childhood morbidity and mortality. Incomplete growth and greater plasticity of juvenile bone means that porous lesions of the cranium may be more likely to develop in skeletally immature individuals, initially affecting the orbital roof and progressing onto the flat bones of the cranium with prolonged exposure to stress^37^. High juvenile mortality may therefore be reflected in the higher prevalence of cribra orbitalia amongst immature skeletal individuals, who died before lesions could manifest on the cranial vault, while high prevalence of porotic hyperostosis amongst adults may signal significant juvenile morbidity in those who managed to survive for a longer period.

Average onset of enamel hypoplasia formation for all populations fell between two and four years of age, corresponding with stable isotope evidence for weaning after two or three years in LBK populations^9^. Weaning may be a stressful period for infants, particularly if weaning food is low in nutrients and softened with water contaminated by pathogens^38^, and although a direct correlation between weaning and hypoplasia formation cannot be attested^39^,^40^, an association is generally postulated^41^.

Later average age at onset of enamel hypoplasia formation in western LBK juveniles could indicate a practice of later weaning or that those individuals weaned early did not survive to six or seven years of age, when the permanent dentition begins to erupt^42^. Weaning may be delayed in modern populations if food is scarce^43^, as may have been the case in the western LBK. Such a delay may correspond with an increase in the length of the inter-birth interval and hence overall a reduction in the rate of population growth within western LBK populations, which is contrary to findings from other cemetery assemblages of an increasing rate of population growth after the adoption of agriculture^44^. Prolonged breastfeeding does not adequately meet the nutritional demands of infants and can result in iron-deficiency and megaloblastic anemia, both of which may lead to the formation of porotic hyperostosis and cribra orbitalia^20^. Any individual weaned early in the western LBK may have been subject to even greater stress than those weaned later if the availability of weaning foods was restricted. As a result, these individuals may have been more likely to develop cribra orbitalia at a young age but then die prior to the eruption of the permanent dentition. Thus high prevalence of porotic hyperostosis and cribra orbitalia but lower juvenile prevalence and later average onset of linear enamel hypoplasia in the western LBK may all signal greater nutritional stress resulting from lower agricultural productivity and different practices of weaning than eastern counterparts.

## Conclusions

Examination of three non-specific indicators of biological stress in five populations from across the east-west distribution of the LBK suggests that the experience of stress within this archaeological culture differed significantly, despite shared characteristics of material culture and population genetics. Porotic hyperostosis and cribra orbitalia were found at higher prevalence within populations from the western region of the LBK, an area associated with lower agricultural productivity, a greater reliance on wild resources, and a diet lower in animal protein. Exposure to stress therefore seems to have been greater towards the western reaches of the LBK and a practice of delayed weaning, possibly as a response to the reduced availability of dietary resources, may have contributed towards this increased morbidity. These results suggest that treating the LBK as a single homogeneous unit may, in fact, be masking a significant diversity of behaviour, which may have important consequences for our understanding of early farming communities.

## Materials and Methods

### Skeletal Material

**Figure 1:**
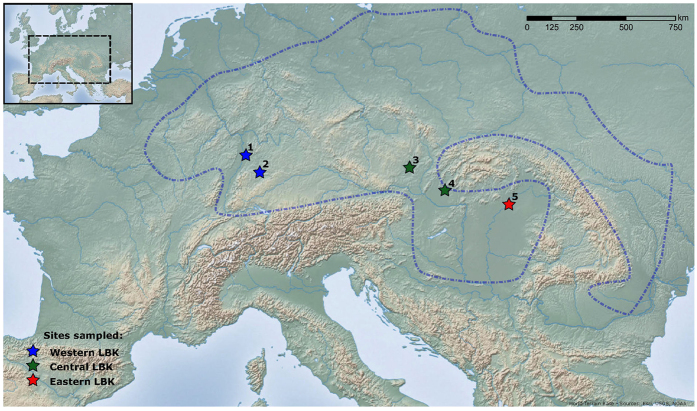
Location map for populations used in this study, showing the regional grouping of sites. See Supplementary Table S2 for more detail. 1 - Schwetzingen; 2 - Stuttgart-Mühlhausen; 3 - Vedrovice; 4 - Nitra-Horné Krškany; 5 - Polgár-Ferenci-hát. World Terrain Base map ( http://tiles.arcgis.com/tiles/jIL9msH9OI208GCb/arcgis/rest/services/World_Relief_Map/MapServer) from ArcGIS [version 10.1], ( http://www.esri.com/software/arcgis).

**Figure 2:**
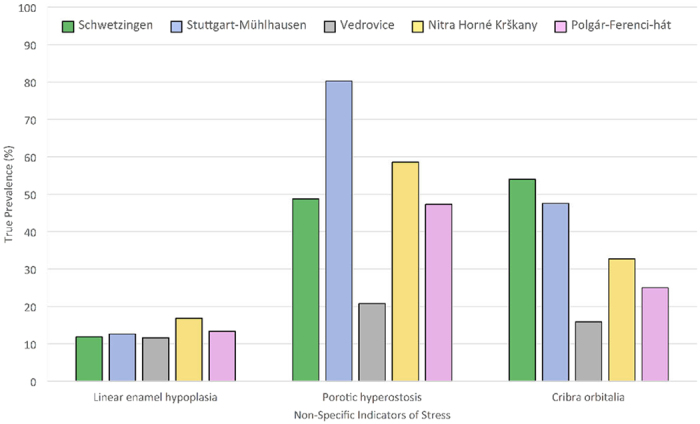
True prevalence of non-specific indicators of stress within the five LBK populations. Populations are arranged in geographic order progressing from west to east. Cribra orbitalia may be seen to be at significantly higher prevalence within both of the populations from the western LBK, while porotic hyperostosis was also found at high prevalence at the western site of Stuttgart-Mühlhausen.

**Figure 3:**
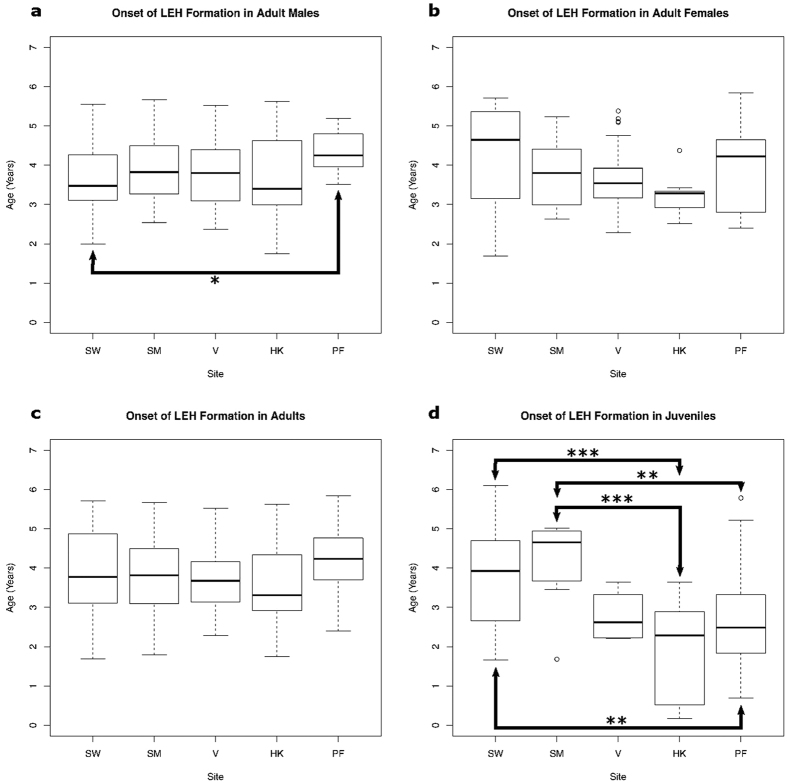
Boxplots of variation in age at onset of hypoplasia formation within each population. Populations are arranged geographically proceeding from west to east. Connecting arrows indicate statistically significant differences between paired populations (*p < 0.02, **p < 0.01, ***p < 0.001). Pairwise comparisons with a p-value of 0.02 or greater are not considered statistically significant due to the high probability of this value occurring by chance when all five pairs are compared. All significant differences involve one or both of the populations from the western LBK. In the juvenile subset, both populations from the western LBK show a later average onset than the more easterly populations. SW - Schwetzingen; SM - Stuttgart-Mühlhausen; V - Vedrovice; HK - Nitra-Horné Krškany; PF - Polgár-Ferenci-hát.

Skeletal remains from five LBK cemeteries were included in this study: the eastern Alföld Pottery site of Polgár-Ferenci-hát; the two sites of Vedrovice and Nitra-Horné Krškany from the central region of the LBK distribution; and Stuttgart-Mühlhausen and Schwetzingen from the western limits of the initial LBK expansion (Fig. 1, Supplementary Table S2). Due to the antiquity of the skeletal material, ethical consent for study was not necessary. The age and sex of each specimen was assessed using standard osteological methods from observation of both the crania and *ossa coxae* where possible^45^,^46^,^47^,^48^,^49^.

A total of 511 skeletons and 2322 teeth, from both the maxilla and mandible, were examined (Table 1). Only the permanent incisors and canines were included in the analysis due to the higher frequency of hypoplasia formation^50^,^51^, and the greater precision with which the location of defects may be measured, on the enamel surface of these single-cusped teeth.

### Macroscopic Analysis

Crania and teeth were examined macroscopically for the presence of each non-specific indicator of stress, and the true prevalence of porotic hyperostosis, cribra orbitalia and linear enamel hypoplasia was calculated as the proportion of the total crania or teeth examined that displayed pathological alterations. In addition to prevalence data, the distance of each hypoplastic defect from the cemento-enamel junction (CEJ) to the border of the defect closest to the tooth cusp was measured. Digital sliding calipers with an accuracy of 0.01mm were used and the location of each defect was measured three times to provide an average. CEJ to defect distances were then used to calculate an age at onset of hypoplasia formation using the Goodman and Rose^18^ equation and tooth crown growth standards from Smith^42^.

### Statistical Testing

Inter-population comparisons of the true prevalence of each non-specific indicator of stress and the age at onset of hypoplasia formation were conducted using the chi-square test for independence and the Kruskal-Wallis analysis of variance, respectively. Tests were conducted between total populations and between the demographic components of each population (adults, juveniles, adult males, and adult females). These analyses tested whether variation in prevalence and onset was random across the dataset or related to the populations being examined. Application of a post-hoc pairwise Wilcoxon rank sum test with Bonferroni adjustment to the results of the Kruskal-Wallis analysis highlighted differences between the median age at onset of paired populations that exceeded the standard error. Comparison of adults and juveniles, and also of adult males and females, using unpaired Wilcoxon rank sum tests further revealed any differences in onset within populations^52^. All statistical testing was conducted using R software^53^.

### Isotopic Analysis

**Figure 4:**
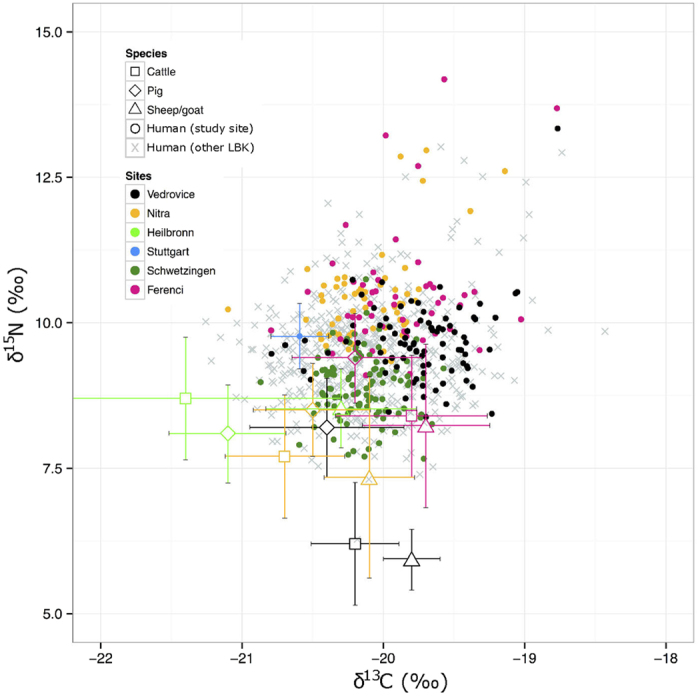
Distribution of carbon and nitrogen stable isotope ratios for LBK populations. Individual isotope values are available in the Supplementary Information (Supplementary Table S6). Stuttgart-Mühlhausen is rep-resented by an average value and standard deviation as full data has not yet been published. Faunal isotope ratios for each site are represented by a mean value bracketed within one standard deviation (see Supplementary Table S7). No faunal data was available for Schwetzingen or Stuttgart-Mühlhausen: fauna from the contemporaneous site of Heilbronn-Neckargartach are plotted as a proxy. Sampled sites show clear groupings which are statistically different. Particularly noticeable are the lower δ^15^N values of human remains from Schwetzingen. Data collected from published sources^8^,^9^,^10^,^11^,^21^,^22^,^27^,^54^,^55^,^56^,^57^ (see Supplementary Tables S6 and S7 and Methods section for full detail).

Data on carbon and nitrogen stable isotope ratios for LBK sites were collected from a review of published literature^8^,^9^,^10^,^11^,^21^,^22^,^27^,^54^,^55^,^56^,^57^. All studies extracted collagen from ribs and long bones (see Supplementary Table S6) using the modified Longin^58^,^59^ method of sample preparation. Ribs are sampled preferentially for analysis of dietary isotopes as they should represent food consumed closer to the time of death of the individual compared to long bones, due to the generally higher rate of bone turnover in ribs^9^, however collagen from ribs and long bones may be comparable to each other while isotopic values from dental collagen are not comparable to either. For this reason published data on dietary isotopes based on dental collagen values were not included in the present analysis. Small differences may arise in carbon and nitrogen isotopic values between sites due to the processing of material at different laboratories, but the use of comparable standards should minimise this confounding factor. Only data for Schwetzingen, Vedrovice, Nitra-Horné Krškany, and Polgár-Ferenci-hát were included in statistical comparisons, all other data from additional sites formed background noise for Fig. 4 but were not tested statistically. Values were plotted using R software^53^ and compared between populations through application of the Kruskal-Wallis analysis of variance statistical test and post-hoc pairwise Wilcoxon rank sum test with Bonferroni adjustment. These tests were performed as the data were determined to be non-normal in distribution. Isotopic values for adult males and females, and also for adults and juveniles, were further compared using two-group Mann-Whitney U tests.

## Additional Information

**How to cite this article**: Ash, A. *et al*. Regional differences in health, diet and weaning patterns amongst the first Neolithic farmers of central Europe. *Sci. Rep.* **6**, 29458; doi: 10.1038/srep29458 (2016).

## Supplementary Material

Supplementary Information

## Figures and Tables

**Table 1:** Summary demographic information for each of the five skeletal collections.

**Site**	**Adults**		**Juveniles**		**Males**		**Females**		**Total**	
	**Ind.**	**Dent.**	**Ind.**	**Dent.**	**Ind.**	**Dent.**	**Ind.**	**Dent.**	**Ind.**	**Dent.**
Schwetzingen	66	498	34	122	35	318	24	180	*100*	*620*
Stuttgart-Mühlhausen	87	421	29	82	49	268	26	129	*116*	*503*
Vedrovice	81	543	30	48	33	272	33	243	*111*	*591*
Nitra-Horné Krškany	53	170	25	36	25	107	20	63	*78*	*206*
Polgár-Ferenci-hát	75	330	31	72	44	191	21	132	*106*	*402*
	*362*	*1962*	*149*	*360*	*186*	*1156*	*124*	*747*	*511*	*2322*

Ind. - number of individual skeletons included in analysis; Dent. - number of teeth.

**Table 2:** Results of chi-square tests comparing true prevalence of linear enamel hypoplasia, porotic hyperostosis and cribra orbitalia between adults and juveniles within each population, and also between adult males and females within each population.

**Indicator**	**Site**	**True Prevalence**												**True Prevalence**								
		**Total**			**Males**			**Females**			**Within population comparison**			**Adults**			**Juveniles**			**Within population comparison**		
		**n**	**n**_**a**_	**%**	**n**	**n**_**a**_	**%**	**n**	**n**_**a**_	**%**	**χ^2^**	**df**	**p**	**n**	**n**_**a**_	**%**	**n**	**n**_**a**_	**%**	**χ^2^**	**df**	**p**
Linear enamel hypoplasia	**SW**	608	72	*11.84*	318	28	*8.81*	180	22	*12.22*	0.31	1	0.577	498	50	*10.04*	122	22	*18.03*	2.03	1	0.155
	**SM**	499	63	*12.63*	268	35	*13.06*	129	11	*8.53*	0.65	1	0.421	421	53	*12.59*	82	10	*12.20*	0.00	1	1.000
	**V**	587	68	*11.58*	272	32	*11.76*	243	27	*11.11*	0.00	1	1.000	543	65	*11.97*	48	3	*6.25*	1.35	1	0.246
	**HK**	238	40	*16.81*	107	12	*11.21*	63	10	*15.87*	0.57	1	0.450	170	22	*12.94*	36	18	*50.00*	30.14	1	**0.000**
	**PF**	390	52	*13.33*	191	10	*5.24*	132	17	*12.88*	2.68	1	0.102	330	27	*8.18*	72	25	*34.72*	19.36	1	**0.000**
Porotic hyperostosis	**SW**	80	39	*48.75*	32	21	*65.63*	20	11	*55.00*	1.94	1	0.164	53	32	*60.38*	27	7	*25.93*	22.81	1	**0.000**
	**SM**	86	69	*80.23*	40	35	*87.50*	16	14	*87.50*	0.00	1	1.000	62	55	*88.71*	24	14	*58.33*	22.16	1	**0.000**
	**V**	82	17	*20.73*	26	7	*26.92*	29	4	*13.79*	4.54	1	0.033	60	13	*21.67*	22	4	*18.18*	0.19	1	0.660
	**HK**	70	41	*58.57*	23	17	*73.91*	17	11	*64.71*	1.58	1	0.208	45	32	*71.11*	25	9	*36.00*	23.39	1	**0.000**
	**PF**	74	35	*47.30*	26	19	*73.08*	18	8	*44.44*	15.75	1	**0.000**	47	29	*61.70*	27	6	*22.22*	30.40	1	**0.000**
Cribra orbitalia	**SW**	63	34	*53.97*	30	16	*53.33*	20	7	*35.00*	6.09	1	**0.014**	50	23	*46.00*	13	11	*84.62*	31.23	1	**0.000**
	**SM**	61	29	*47.54*	30	15	*50.00*	13	3	*23.08*	14.49	1	**0.000**	46	19	*41.30*	15	10	*66.67*	11.95	1	**0.001**
	**V**	63	10	*15.87*	23	4	*17.39*	25	2	*8.00*	3.18	1	0.075	49	6	*12.24*	14	4	*28.57*	7.23	1	**0.007**
	**HK**	55	18	*32.73*	22	4	*18.18*	13	6	*46.15*	16.67	1	**0.000**	36	11	*30.56*	19	7	*36.84*	0.63	1	0.429
	**PF**	52	13	*25.00*	18	1	*5.56*	17	6	*35.29*	25.41	1	**0.000**	35	7	*20.00*	17	6	*35.29*	5.11	1	0.024

Prevalence represented the observed value for each demographic grouping and the mid-point of these was taken as the expected value for each separate test. Statistically significant results are highlighted in bold font. Due to the large number of tests performed some statistical significance is expected to occur by chance. To combat this, only tests returning a p value less than 0.02 were considered significant. SW - Schwetzingen; SM - Stuttgart-Mühlhausen; V - Vedrovice; HK - Nitra-Horné Krškany; PF - Polgár-Ferenci-hát; n - sample size; n_a_ - number of sample affected by indicator; % - prevalence of indicator in sample.

**Table 3:** Results of chi-square tests comparing true prevalence of linear enamel hypoplasia, porotic hyperostosis and cribra orbitalia between the five populations.

**Category**	**Linear enamel hypoplasia**			**Porotic hyperostosis**			**Cribra orbitalia**		
	**χ^2^**	**df**	**p**	**χ^2^**	**df**	**p**	**χ^2^**	**df**	**p**
Total	1.55	4	0.818	73.91	4	**0.000**	43.42	4	**0.000**
Males	4.22	4	0.376	92.83	4	**0.000**	89.29	4	**0.000**
Females	2.68	4	0.612	118.12	4	**0.000**	40.61	4	**0.000**
Adults	1.62	4	0.806	101.36	4	**0.000**	38.05	4	**0.000**
Juveniles	69.74	4	**0.000**	47.36	4	**0.000**	92.96	4	**0.000**

Prevalence at each site represented the observed value while expected values for each separate test were estimated as the mean prevalence of all five sites. Significant variability in the prevalence of porotic hyperostosis and cribra orbitalia is evident between sites. Statistically significant results are highlighted in bold font.

**Table 4:** Average age at onset of linear enamel hypoplasia formation for each population (total) and for each demographic grouping within populations.

**Site**	**Adults**	**Juveniles**	**Males**	**Females**	**Total**
Schwetzingen	4.0	3.8	3.7	4.4	3.9
Stuttgart-Mühlhausen	3.8	4.2	3.9	3.7	3.9
Vedrovice	3.7	2.8	3.8	3.6	3.7
Nitra-Horné Krškany	3.6	1.9	3.7	3.2	2.9
Polgár-Ferenci-hát	4.2	2.7	4.4	4.0	3.2

All ages are in years. Only juveniles show significant variation in average age at onset between populations, being later in western than eastern populations. See Supplementary Tables S4 and S5 for statistical significance of differences.
