# Supplementary material for: Regional differences in health, diet and weaning patterns amongst the first Neolithic farmers of central Europe
Source: Sci Rep. 2016 Jul 7;6:29458. doi: 10.1038/srep29458 (PMC4935844; doi:10.1038/srep29458)
Supplement: Supplementary Information [file srep29458-s1.pdf]

Supplementary Information

Regional differences in health, diet and weaning patterns amongst the first Neolithic farmers of central Europe.

Abigail Ash[a]*, Michael Francken[b], Ildikó Pap[c], Zdeněk Tvrdý[d], Joachim Wahl[e,f] & Ron Pinhasi[a,g]

[a] School of Archaeology, University College Dublin, Dublin 4, Republic of Ireland
[b] Institute for Archaeological Sciences and Senckenberg Center for Human Evolution and Paleoenvironment, University of Tübingen, Germany
[c] Department of Anthropology, Hungarian Natural History Museum, Budapest, Hungary
[d] Anthropos Institute, Moravian Museum, Zelný trh 6, Brno, Czech Republic
[e] State Office for Cultural Heritage Management Baden-Württemberg, Osteology, D-78467 Konstanz, Germany
[f] Institute for Archaeological Sciences, WG Palaeoanthropology, University of Tübingen, Germany
[g] Earth Institute, University College Dublin, Dublin 4, Republic of Ireland
* abigail.ash@ucd.ie

Supplementary Table S1: Comparison of non-specific stress in LBK populations from this study and from previously published sources. All three markers of non-specific stress were not always recorded for previously published reports, however a higher prevalence of cribra orbitalia may be seen at Austrian sites than those further east in Hungary, matching the results of the present study. Prevalence of porotic hyperostosis and linear enamel hypoplasias for all the populations reported here were higher than previously recorded. This may relate to differences in the diagnostic criteria of pathological lesions between studies and also differences in the range of teeth included in analyses. Brackets indicate approximate dating of the time period with which the site is associated. [a] Reported as crude prevalence: the proportion of individuals affected from the total number of individuals in the collection.

Site Information			Prevalence			Reference
Name	Location	Date	Porotic hyperostosis	Cribra orbitalia	Linear enamel hypoplasia	
Ensisheim les Octrois	Haute-Alsace	(5200-4900 cal. BC)		6.70%	2.10%	1
Schwetzingen	Germany	5260-5010 cal. BC	48.75%	53.97%	11.84%	This study
Stuttgart-Mühlhausen	Germany	5200-4960 cal.BC	80.23%	47.54%	12.63%	This study
Asparn an der Zaya/Schletz	Austria	5210-4950 cal. BC		55.22%[a]		2
Mitterndorf im Tullnerfeld	Austria	(5200-5000 cal. BC)		25%	3.20%	2
Rutzing	Austria	(5200-5000 cal. BC)	~1%[a]	55.60%	3.60%	2
Nitra-Horné-Krškany	Slovakia	5370-4980 cal. BC	1.33%[a]	15%	3.80%	3
Nitra-Horné-Krškany	Slovakia	5370-4980 cal. BC	58.57%	33%	16.81%	This study
Vedrovice	Czech Republic	5300-5100 cal. BC		14.58%	6.17%[a]	4
Vedrovice	Czech Republic	5300-5100 cal. BC	20.73%	15.87%	11.58%	This study
Füzesabony-Gubakút	Hungary	5325-5220 cal. BC		12.20%	4.60%	5
Polgár-Ferenci-hát	Hungary	5300-5070 cal. BC		12.20%	12.40%	5
Polgár-Ferenci-hát	Hungary	5300-5070 cal. BC	47.30%	25.00%	13.33%	This study

Supplementary Table S2: Brief archaeological exploration of populations used in this study. Cemetery size is indicated by the number of burials. Where the number of burials differs from the number of individuals included in this study, this reflects random sampling due to restrictions on time and resources, inability to locate specimens and/or inclusion of cremation burials within the total. *The cemetery at Stuttgart-Mühlhausen contained 247 burials, but only 177 could be dated to the Early Neolithic based on associated ceramic goods, the sample included in this study was drawn from these graves.

Region	Site	Date	Environment	Diet	Burials	Cemetery Size
**Western**	Schwetzingen	5260-5010 cal. BC	Loess sediment on east bank of River Rhine	Mixed terrestrial, cattle and pig, high in wild fauna, predominantly wheat based	Cemetery - single, left-side flexed, NE-SW orientation, 50% with goods	218
	Stuttgart-Mühlhausen	5200-4960 cal. BC	Loess sediment in Neckar Valley		Cemetery -single, right-side flexed, W-E orientation, >50% with goods	247*
**Central**	Nitra Horné Krškany	5370-4980 cal. BC	Floodplain loess plateau on Nitra River	Mixed terrestrial, cattle dominated, predominantly wheat based	Cemetery -single, left-side flexed, SE-NW orientation, 79% with goods	81
	Vedrovice	5300-5100 cal. BC	Loess sediment, near several small rivers		Cemetery and settlement - single, left-side flexed, E-W orientation, 73% with goods	111
**Eastern**	Polgár-Ferenci-hát	5300-5070 cal. BC	Loess alluvial island south of River Tisza	Mixed terrestrial, cattle and sheep/goat, wheat and barley based	Settlement - single, left-side flexed, SE-NW orientation, 28% with goods	117

Supplementary Table S3: Demographic and palaeopathological data for the skeletons included in this paper. LEH - linear enamel hypoplasia; PH - porotic hyperostosis; CO - cribra orbitalia.

**Site**	**Skeletal ID**	**LBK Region**	**Age range**	**Sex**	**LEH**	**PH**	**State of PH**	**CO**	**State of CO**
Nitra-Horné Krškany	obj3	Central	Adult	?	N	**Y**	Healed	N	
Nitra-Horné Krškany	S3	Central	Adult	?	N	**Y**	Healed	N	
Nitra-Horné Krškany	S8	Central	Adult	?	N	N		N	
Nitra-Horné Krškany	01	Central	Adult	Female	N	N		N	
Nitra-Horné Krškany	02	Central	Adult	Male	N	N		N	
Nitra-Horné Krškany	03	Central	14-15yrs	Male	N	**Y**	Healed	N	
Nitra-Horné Krškany	04	Central	40-45yrs	Male	N	N		N	
Nitra-Horné Krškany	04A	Central	25-35yrs	Female	N	N		N	
Nitra-Horné Krškany	04B	Central	7-13yrs	?	N	N		N	
Nitra-Horné Krškany	05	Central	16-17yrs	Male	N	N		**Y**	Active
Nitra-Horné Krškany	06	Central	35-45yrs	Female	N	**Y**	Healed	N	
Nitra-Horné Krškany	07	Central	20-25yrs	Male	N	**Y**	Healed	N	
Nitra-Horné Krškany	08	Central	35-45yrs	Male	N	**Y**	Healed	N	
Nitra-Horné Krškany	09	Central	46-59yrs	?	N	**Y**	Active	**Y**	Active
Nitra-Horné Krškany	13A	Central	5-6yrs	Male	N	**Y**	Healed	N	
Nitra-Horné Krškany	13B	Central	8-9yrs	Male	**Y**	**Y**	Healed	N	
Nitra-Horné Krškany	14	Central	Adult	?	N	N		N	
Nitra-Horné Krškany	15	Central	Adult	?	N	N		N	
Nitra-Horné Krškany	16	Central	Adult	?	N	**Y**	Healed	N	
Nitra-Horné Krškany	17	Central	50-55yrs	Male	N	**Y**	Healed	N	
Nitra-Horné Krškany	18	Central	Adult	Female	N	N		**Y**	Healed
Nitra-Horné Krškany	19	Central	25-35yrs	Male	N	**Y**	Healed	N	
Nitra-Horné Krškany	20	Central	25-30yrs	Female	N	N		**Y**	Healed
Nitra-Horné Krškany	21	Central	50-55yrs	Male	N	N		N	
Nitra-Horné Krškany	22	Central	50-55yrs	Male	N	N		N	
Nitra-Horné Krškany	23	Central	12-14yrs	Male	**Y**	N		N	
Nitra-Horné Krškany	24	Central	30-35yrs	Female	**Y**	**Y**	Healed	N	
Nitra-Horné Krškany	25	Central	45-50yrs	Male	N	**Y**	Healed	N	
Nitra-Horné Krškany	26	Central	30-35yrs	Male	N	**Y**	Healed	N	
Nitra-Horné Krškany	27	Central	50-55yrs	Female	N	N		**Y**	Healed
Nitra-Horné Krškany	28	Central	9-12m	Female	N	N		N	
Nitra-Horné Krškany	29	Central	7-13yrs	?	N	**Y**	Healed	**Y**	Active
Nitra-Horné Krškany	30	Central	1-3yrs	Female	N	**Y**	Active	N	
Nitra-Horné Krškany	31	Central	1-3yrs	Male	N	N		**Y**	Healed
Nitra-Horné Krškany	32	Central	16-18yrs	Female	N	**Y**	Healed	**Y**	Healed
Nitra-Horné Krškany	33	Central	18-20yrs	Male	N	**Y**	Healed	**Y**	Healed
Nitra-Horné Krškany	34	Central	30-35yrs	Male	N	**Y**	Healed	N	
Nitra-Horné Krškany	35	Central	40-45yrs	Male	N	N		**Y**	Healed
Nitra-Horné Krškany	36	Central	45-50yrs	Male	N	**Y**	Healed	N	
Nitra-Horné Krškany	37	Central	30-35yrs	Female	**Y**	**Y**	Healed	**Y**	Healed
Nitra-Horné Krškany	38	Central	6-7yrs	Male	N	**Y**	Healed	N	
Nitra-Horné Krškany	38Z	Central	Adult	?	N	N		N	
Nitra-Horné Krškany	39	Central	45-50yrs	Male	N	**Y**	Healed	**Y**	Healed
Nitra-Horné Krškany	40	Central	1-6yrs	?	N	N		N	
Nitra-Horné Krškany	40Z	Central	Adult	?	N	N		N	
Nitra-Horné Krškany	41	Central	12-14yrs	Female	N	N		N	

Nitra-Horné Krškany	42	Central	Juvenile	?	N	N		N	
Nitra-Horné Krškany	43	Central	Adult	Female	N	N		N	
Nitra-Horné Krškany	44	Central	30-35yrs	Male	**Y**	**Y**	Healed	N	
Nitra-Horné Krškany	45	Central	Adult	Male	N	**Y**	Healed	N	
Nitra-Horné Krškany	46	Central	Adult	?	N	N		N	
Nitra-Horné Krškany	47	Central	1-6yrs	?	N	N		N	
Nitra-Horné Krškany	48	Central	18-20yrs	Male	**Y**	**Y**	Healed	N	
Nitra-Horné Krškany	49	Central	7-13yrs	Male	N	N		N	
Nitra-Horné Krškany	50	Central	Juvenile	?	N	N		**Y**	Active
Nitra-Horné Krškany	52	Central	35-45yrs	Female	N	**Y**	Healed	N	
Nitra-Horné Krškany	53	Central	18-20yrs	Female	**Y**	**Y**	Healed	**Y**	Healed
Nitra-Horné Krškany	54	Central	6-7yrs	Female	N	N		N	
Nitra-Horné Krškany	55	Central	1-3m	Female	N	N		N	
Nitra-Horné Krškany	56	Central	40-45yrs	Male	N	**Y**	Healed	N	
Nitra-Horné Krškany	57	Central	20-25yrs	Female	N	**Y**	Healed	N	
Nitra-Horné Krškany	58	Central	25-35yrs	Male	N	N		N	
Nitra-Horné Krškany	59	Central	13-15yrs	Male	**Y**	**Y**	Healed	**Y**	Healed
Nitra-Horné Krškany	60	Central	1-12m	?	N	N		N	
Nitra-Horné Krškany	61	Central	35-40yrs	Female	N	**Y**	Healed	N	
Nitra-Horné Krškany	62	Central	Adult	Female	N	N		N	
Nitra-Horné Krškany	63	Central	Adult	Male	N	N		N	
Nitra-Horné Krškany	64	Central	35-40yrs	Female	N	N		N	
Nitra-Horné Krškany	65	Central	25-30yrs	Female	N	**Y**	Healed	N	
Nitra-Horné Krškany	66	Central	40-45yrs	Female	N	**Y**	Healed	N	
Nitra-Horné Krškany	67	Central	4-5yrs	Female	N	N		N	
Nitra-Horné Krškany	68	Central	Adult	Female	N	**Y**	Healed	N	
Nitra-Horné Krškany	69	Central	18-20yrs	Male	**Y**	**Y**	Healed	N	
Nitra-Horné Krškany	70	Central	25-35yrs	Female	N	**Y**	Healed	**Y**	Healed
Nitra-Horné Krškany	71	Central	2-3yrs	Male	N	**Y**	Healed	N	
Nitra-Horné Krškany	72	Central	25-30yrs	Male	**Y**	**Y**	Active	**Y**	Healed
Nitra-Horné Krškany	73	Central	6m	?	N	N		N	
Nitra-Horné Krškany	74	Central	4-5yrs	Male	N	N		**Y**	Healed
Nitra-Horné Krškany	75	Central	Adult	?	N	N		N	
Nitra-Horné Krškany	76	Central	Adult	Male	N	**Y**	Healed	N	
Nitra-Horné Krškany	77	Central	Adult	Male	N	N		N	
Polgár-Ferenci-hát	103/208	Eastern	30-35 y	Male	**Y**	N		N	
Polgár-Ferenci-hát	104/209	Eastern	6-9 months	?	N	N		N	
Polgár-Ferenci-hát	118/223	Eastern	30-35y	Female	**Y**	N		N	
Polgár-Ferenci-hát	126/231	Eastern	35-40y	Male	N	**Y**	Healed	N	
Polgár-Ferenci-hát	134/239	Eastern	16-18y	Male	**Y**	N		N	
Polgár-Ferenci-hát	144/252	Eastern	6-9m	Male	N	N		N	
Polgár-Ferenci-hát	145/253	Eastern	1.5-2y	Male	N	N		**Y**	Healed
Polgár-Ferenci-hát	21/75	Eastern	25-30y	Male	N	N		N	
Polgár-Ferenci-hát	280/443	Eastern	30-35y	Male	N	**Y**	Healed	N	
Polgár-Ferenci-hát	281/444	Eastern	40-45y	Female	**Y**	N		N	
Polgár-Ferenci-hát	283/448	Eastern	8-9y	?	N	N		N	
Polgár-Ferenci-hát	284/449	Eastern	46-59y	Male	N	N		N	
Polgár-Ferenci-hát	285/450	Eastern	30-35y	Male	N	N		N	
Polgár-Ferenci-hát	288/453	Eastern	1.5-2y	Male	N	N		**Y**	Healed
Polgár-Ferenci-hát	289/454	Eastern	8-9y	Male	N	N		N	
Polgár-Ferenci-hát	291/457	Eastern	46-59y	Male	N	N		N	
Polgár-Ferenci-hát	296/468	Eastern	25-30y	Female	N	N		N	

Polgár-Ferenci-hát	31/33	Eastern	25-30y	Male	**Y**	**Y**	Healed	N	
Polgár-Ferenci-hát	311/486	Eastern	35-40y	Female	N	N		N	
Polgár-Ferenci-hát	312/487	Eastern	35-40y	Female	N	N		N	
Polgár-Ferenci-hát	313/488	Eastern	46-59y	?	N	**Y**	Healed	N	
Polgár-Ferenci-hát	318/493	Eastern	4-5y	?	N	N		N	
Polgár-Ferenci-hát	325/500	Eastern	12-14y	Male	**Y**	N		N	
Polgár-Ferenci-hát	337/516	Eastern	Adult	?	N	N		N	
Polgár-Ferenci-hát	338/517	Eastern	12-14y	Male	**Y**	N		N	
Polgár-Ferenci-hát	339/518	Eastern	30-35y	Male	N	N		N	
Polgár-Ferenci-hát	34/82	Eastern	35-40y	Female	N	**Y**	Healed	N	
Polgár-Ferenci-hát	34/91	Eastern	10-12y	Female	**Y**	**Y**	Healed	**Y**	Healed
Polgár-Ferenci-hát	340/519	Eastern	30-35y	Male	N	**Y**	Healed	N	
Polgár-Ferenci-hát	341/520	Eastern	25-30y	Male	N	**Y**	Healed	N	
Polgár-Ferenci-hát	342/521	Eastern	46-59y	Female	N	**Y**	Healed	N	
Polgár-Ferenci-hát	344/523	Eastern	18-25y	Female	N	**Y**	Healed	N	
Polgár-Ferenci-hát	345/524	Eastern	20-25y	Female	**Y**	N		**Y**	Healed
Polgár-Ferenci-hát	348/527	Eastern	6-9m	Female	N	N		N	
Polgár-Ferenci-hát	349/528	Eastern	1-1.5y	Male	N	N		N	
Polgár-Ferenci-hát	350/529	Eastern	5-6y	Female	N	N		N	
Polgár-Ferenci-hát	351/530	Eastern	9-10y	Male	N	**Y**	Healed	N	
Polgár-Ferenci-hát	352/531	Eastern	30-35y	Female	N	N		N	
Polgár-Ferenci-hát	353/532	Eastern	35-45y	Male	N	N		N	
Polgár-Ferenci-hát	354/533	Eastern	Adult	Female	N	N		N	
Polgár-Ferenci-hát	356/535	Eastern	35-45y	Male	N	N		N	
Polgár-Ferenci-hát	357/536	Eastern	35-40y	Male	N	**Y**	Healed	N	
Polgár-Ferenci-hát	360/545	Eastern	Adult	?	N	N		N	
Polgár-Ferenci-hát	361/549	Eastern	25-30y	?	N	N		N	
Polgár-Ferenci-hát	362/550	Eastern	40-45y	Male	N	N		N	
Polgár-Ferenci-hát	363/551	Eastern	Adult	?	N	N		N	
Polgár-Ferenci-hát	364/552	Eastern	25-30y	Male	N	N		N	
Polgár-Ferenci-hát	368/562	Eastern	25-30y	Female	N	N		N	
Polgár-Ferenci-hát	387/581	Eastern	30-35y	Male	N	N		N	
Polgár-Ferenci-hát	4/4	Eastern	40-45y	Male	N	**Y**	Healed	N	
Polgár-Ferenci-hát	41/74	Eastern	2-3y	Female	N	N		N	
Polgár-Ferenci-hát	416/630	Eastern	Adult	Male	N	N		N	
Polgár-Ferenci-hát	448/646	Eastern	2-3y	?	N	N		N	
Polgár-Ferenci-hát	449/647	Eastern	Adult	Male	N	**Y**	Healed	N	
Polgár-Ferenci-hát	451/722	Eastern	46-59y	Male	N	**Y**	Healed	N	
Polgár-Ferenci-hát	481/858	Eastern	7-13y	?	N	**Y**	Healed	N	
Polgár-Ferenci-hát	484/685	Eastern	12-14y	Male	N	N		N	
Polgár-Ferenci-hát	486/687	Eastern	30-35y	Male	N	N		N	
Polgár-Ferenci-hát	499/701	Eastern	Adult	Male	N	N		N	
Polgár-Ferenci-hát	510/711	Eastern	Adult	?	N	**Y**	Healed	N	
Polgár-Ferenci-hát	534/748	Eastern	Adult	Male	N	N		N	
Polgár-Ferenci-hát	546/763	Eastern	Adult	?	N	N		N	
Polgár-Ferenci-hát	552/770	Eastern	36-45y	Male	N	N		N	
Polgár-Ferenci-hát	6/7	Eastern	Adult	?	N	N		N	
Polgár-Ferenci-hát	604/865	Eastern	36-45y	Male	N	N		N	
Polgár-Ferenci-hát	636/930	Eastern	46-59y	Male	N	**Y**	Healed	N	
Polgár-Ferenci-hát	640/933	Eastern	2-3y	?	N	N		N	
Polgár-Ferenci-hát	641/987	Eastern	30-35y	Male	N	N		N	
Polgár-Ferenci-hát	644/944	Eastern	30-35y	Male	N	**Y**	Healed	N	

Polgár-Ferenci-hát	691/998	Eastern	40-45y	Male	N	**Y**	Healed	N	
Polgár-Ferenci-hát	697/1004	Eastern	35-40y	Male	N	**Y**	Healed	N	
Polgár-Ferenci-hát	715/1022	Eastern	30-35y	Male	N	N		N	
Polgár-Ferenci-hát	717/1024	Eastern	18-20y	Female	N	N		**Y**	Active
Polgár-Ferenci-hát	718/1025	Eastern	18-20y	Male	N	**Y**	Healed	N	
Polgár-Ferenci-hát	719/1026	Eastern	2-3y	Male	N	**Y**	Healed	**Y**	Healed
Polgár-Ferenci-hát	721/1028	Eastern	30-35y	Male	N	N		N	
Polgár-Ferenci-hát	732/1040	Eastern	35-40y	Male	**Y**	**Y**	Healed	N	
Polgár-Ferenci-hát	738/1046	Eastern	35-40y	Male	N	N		N	
Polgár-Ferenci-hát	764/1079	Eastern	25-30y	Male	N	**Y**	Healed	**Y**	Healed
Polgár-Ferenci-hát	768/1084	Eastern	20-25y	Female	**Y**	**Y**	Healed	N	
Polgár-Ferenci-hát	773/1093	Eastern	40-45y	Female	**Y**	**Y**	Healed	N	
Polgár-Ferenci-hát	782/1105	Eastern	20-25y	Male	N	**Y**	Healed	N	
Polgár-Ferenci-hát	786/1109	Eastern	1-1.5y	Male	N	N		N	
Polgár-Ferenci-hát	801/1130	Eastern	25-30y	Female	N	N		N	
Polgár-Ferenci-hát	805/1138	Eastern	Adult	Male	N	**Y**	Healed	N	
Polgár-Ferenci-hát	806/1139	Eastern	Adult	?	N	N		N	
Polgár-Ferenci-hát	807/1140	Eastern	30-35y	Female	N	**Y**	Healed	**Y**	Healed
Polgár-Ferenci-hát	808/1141	Eastern	2-3y	Female	N	N		N	
Polgár-Ferenci-hát	811/1144	Eastern	4-5y	Male	N	N		**Y**	Healed
Polgár-Ferenci-hát	815/1128	Eastern	6-7y	Male	N	N		N	
Polgár-Ferenci-hát	821/1168	Eastern	30-35y	Female	N	**Y**	Healed	**Y**	Active
Polgár-Ferenci-hát	822/1169	Eastern	1m post	?	N	N		N	
Polgár-Ferenci-hát	824/1171	Eastern	Adult	?	N	N		N	
Polgár-Ferenci-hát	839/1198	Eastern	40-45y	Female	N	N		**Y**	Healed
Polgár-Ferenci-hát	861/1222	Eastern	12-14y	Male	**Y**	**Y**	Healed	N	
Polgár-Ferenci-hát	867/1230	Eastern	25-30y	Male	N	N		N	
Polgár-Ferenci-hát	869/1005	Eastern	Adult	Male	N	N		N	
Polgár-Ferenci-hát	870/1235	Eastern	18-20y	Female	N	N		N	
Polgár-Ferenci-hát	871/1236	Eastern	2-3y	Male	N	**Y**	Healed	N	
Polgár-Ferenci-hát	881/1252	Eastern	2-3y	Female	N	N		N	
Polgár-Ferenci-hát	889/1260	Eastern	20-25y	Female	N	**Y**	Active	**Y**	Active
Polgár-Ferenci-hát	890/1261	Eastern	35-40y	Male	N	N		N	
Polgár-Ferenci-hát	897/1268	Eastern	25-30y	Male	N	**Y**	Healed	N	
Polgár-Ferenci-hát	899/1270	Eastern	35-40y	Male	N	N		N	
Polgár-Ferenci-hát	900/1271	Eastern	1-2y	Female	N	N		**Y**	Active
Polgár-Ferenci-hát	94/179	Eastern	3-6m	?	N	N		N	
Stuttgart-Mühlhausen	(16)	Western	Adult	Female	N	**Y**	Healed	N	
Stuttgart-Mühlhausen	(18)	Western	Adult	Male	N	N		N	
Stuttgart-Mühlhausen	(2)	Western	14-16y	?	N	**Y**	Healed	N	
Stuttgart-Mühlhausen	1046(23)	Western	Adult	?	N	**Y**	Healed	N	
Stuttgart-Mühlhausen	1086(36)	Western	35-40y	Male	N	N		N	
Stuttgart-Mühlhausen	1087(37)	Western	Adult	?	N	N		N	
Stuttgart-Mühlhausen	1088(38)	Western	Adult	Male	N	N		N	
Stuttgart-Mühlhausen	1092(39)	Western	25-30y	Female	N	N		N	
Stuttgart-Mühlhausen	110	Western	Adult	?	N	**Y**	Healed	N	
Stuttgart-Mühlhausen	1240(41)	Western	11-12y	?	**Y**	**Y**	Healed	**Y**	Healed
Stuttgart-Mühlhausen	1242(43)	Western	20-25y	Male	**Y**	**Y**	Healed	**Y**	Healed
Stuttgart-Mühlhausen	1251(44)	Western	18-25y	Male	**Y**	**Y**	Healed	N	
Stuttgart-Mühlhausen	1252(45)	Western	18-25y	Male	N	**Y**	Healed	N	
Stuttgart-Mühlhausen	1253(46)	Western	12-13y	Female	**Y**	N		N	
Stuttgart-Mühlhausen	1254(47)	Western	30-35y	Male	**Y**	**Y**	Healed	N	

Stuttgart-Mühlhausen	1256(48)	Western	46-59y	Male	N	**Y**	Healed	**Y**	Healed
Stuttgart-Mühlhausen	1259(52)	Western	35-45y	Male	N	N		N	
Stuttgart-Mühlhausen	1260(53)	Western	9-10y	?	**Y**	N		**Y**	Healed
Stuttgart-Mühlhausen	1299(54)	Western	35-40y	Female	**Y**	**Y**	Healed	N	
Stuttgart-Mühlhausen	1301(57)	Western	25-30y	Male	**Y**	**Y**	Healed	N	
Stuttgart-Mühlhausen	1348(49)	Western	2-3y	Female	N	N		**Y**	Healed
Stuttgart-Mühlhausen	1428(68)	Western	25-30y	?	**Y**	**Y**	Healed	**Y**	Active
Stuttgart-Mühlhausen	1577(70)	Western	30-35y	Female	**Y**	**Y**	Healed	N	
Stuttgart-Mühlhausen	1578(71)	Western	30-35y	Male	N	**Y**	Healed	N	
Stuttgart-Mühlhausen	1579(72)	Western	2-3y	Female	N	N		N	
Stuttgart-Mühlhausen	1582(76)	Western	Adult	?	N	N		N	
Stuttgart-Mühlhausen	1584(78)	Western	25-30y	Male	**Y**	**Y**	Healed	**Y**	Healed
Stuttgart-Mühlhausen	159(21)	Western	25-30y	Male	N	**Y**	Healed	**Y**	Healed
Stuttgart-Mühlhausen	175(10)	Western	30-35y	Male	N	N		**Y**	Healed
Stuttgart-Mühlhausen	1805(96)	Western	25-30y	Male	**Y**	N		N	
Stuttgart-Mühlhausen	187(5)	Western	Adult	Male	N	**Y**	Healed	N	
Stuttgart-Mühlhausen	195(26)	Western	40-45y	Male	N	**Y**	Healed	N	
Stuttgart-Mühlhausen	1972(102)	Western	35-40y	Male	N	**Y**	Healed	N	
Stuttgart-Mühlhausen	1974(104)	Western	30-35y	Male	N	**Y**	Healed	**Y**	Healed
Stuttgart-Mühlhausen	1976(106)	Western	40-45y	Female	N	**Y**	Healed	N	
Stuttgart-Mühlhausen	1977(107)	Western	20-25y	Male	**Y**	**Y**	Healed	N	
Stuttgart-Mühlhausen	1993(114)	Western	7-9y	Male	**Y**	N		N	
Stuttgart-Mühlhausen	1994(115)	Western	7-8y	Female	N	N		N	
Stuttgart-Mühlhausen	201(15)	Western	Adult	?	N	N		N	
Stuttgart-Mühlhausen	204(12)	Western	4-6y	Female	N	**Y**	Healed	**Y**	Healed
Stuttgart-Mühlhausen	205(22)	Western	35-40y	Male	N	**Y**	Healed	**Y**	Healed
Stuttgart-Mühlhausen	207(25)	Western	25-30y	Male	**Y**	**Y**	Healed	**Y**	Healed
Stuttgart-Mühlhausen	208(20)	Western	30-35y	Male	N	**Y**	Healed	**Y**	Healed
Stuttgart-Mühlhausen	209(32)	Western	35-40y	Male	N	**Y**	Healed	N	
Stuttgart-Mühlhausen	2117(127)	Western	40-45y	Male	N	**Y**	Healed	**Y**	Healed
Stuttgart-Mühlhausen	2119(129)	Western	35-40y	Male	**Y**	**Y**	Healed	N	
Stuttgart-Mühlhausen	2152(122)	Western	Adult	?	N	**Y**	Healed	N	
Stuttgart-Mühlhausen	2154(124)	Western	Juvenile	?	N	N		N	
Stuttgart-Mühlhausen	2170(131)	Western	10-11y	Female	N	**Y**	Healed	N	
Stuttgart-Mühlhausen	2173(134)	Western	Adult	Female	N	N		N	
Stuttgart-Mühlhausen	254(36)	Western	35-40y	Male	N	**Y**	Healed	**Y**	Healed
Stuttgart-Mühlhausen	255(45)	Western	35-40y	Female	N	**Y**	Healed	**Y**	Active
Stuttgart-Mühlhausen	256(37)	Western	30-35y	Female	N	**Y**	Healed	N	
Stuttgart-Mühlhausen	257(35)	Western	10-12y	Male	N	**Y**	Healed	N	
Stuttgart-Mühlhausen	258(47)	Western	30-35y	Male	N	**Y**	Healed	**Y**	Healed
Stuttgart-Mühlhausen	259(49)	Western	12-14y	?	N	**Y**	Healed	**Y**	Active
Stuttgart-Mühlhausen	259(61)	Western	50-60y	Female	N	N		N	
Stuttgart-Mühlhausen	260(48)	Western	30-35y	Male	N	**Y**	Healed	**Y**	Healed
Stuttgart-Mühlhausen	261(40)	Western	35-40y	Male	N	**Y**	Healed	**Y**	Healed
Stuttgart-Mühlhausen	274(27)	Western	30-35y	Female	N	**Y**	Healed	N	
Stuttgart-Mühlhausen	29(24)	Western	20-25y	Female	N	N		N	
Stuttgart-Mühlhausen	3(9)	Western	20-25y	Male	N	**Y**	Healed	N	
Stuttgart-Mühlhausen	332(68)	Western	30-35y	Female	N	**Y**	Healed	**Y**	Active
Stuttgart-Mühlhausen	333(57)	Western	35-45y	?	**Y**	**Y**	Healed	N	
Stuttgart-Mühlhausen	334(70)	Western	18-20y	Male	N	**Y**	Healed	**Y**	Healed
Stuttgart-Mühlhausen	335(64)	Western	40-45y	Male	N	**Y**	Healed	N	
Stuttgart-Mühlhausen	336(54)	Western	Adult	?	N	N		N	

Stuttgart-Mühlhausen	337(65)	Western	30-35y	?	N	**Y**	Healed	N	
Stuttgart-Mühlhausen	337(65a)	Western	0-1m	?	N	N		N	
Stuttgart-Mühlhausen	338(60)	Western	40-45y	Male	N	N		N	
Stuttgart-Mühlhausen	339(67)	Western	40-45y	Male	**Y**	**Y**	Healed	N	
Stuttgart-Mühlhausen	340(72)	Western	46-59y	Male	N	N		N	
Stuttgart-Mühlhausen	341(66)	Western	45-50y	Male	N	N		N	
Stuttgart-Mühlhausen	531(61)	Western	45-50y	Female	N	**Y**	Healed	N	
Stuttgart-Mühlhausen	377(73)	Western	10-12y	Male	N	N		N	
Stuttgart-Mühlhausen	378(76)	Western	Adult	Male	N	N		N	
Stuttgart-Mühlhausen	379(77)	Western	18-20y	Female	N	**Y**	Healed	**Y**	Healed
Stuttgart-Mühlhausen	476(6)	Western	25-30y	Male	N	**Y**	Healed	N	
Stuttgart-Mühlhausen	477(52)	Western	6-7y	Male	N	**Y**	Healed	N	
Stuttgart-Mühlhausen	500(1)	Western	5-6y	Male	N	N		N	
Stuttgart-Mühlhausen	501(2)	Western	16-18y	Female	N	**Y**	Active	N	
Stuttgart-Mühlhausen	502(3)	Western	Adult	?	N	N		N	
Stuttgart-Mühlhausen	503(4)	Western	2-6y	?	N	N		N	
Stuttgart-Mühlhausen	504(7)	Western	20-25y	Female	N	N		N	
Stuttgart-Mühlhausen	505(8)	Western	46-59y	Female	N	N		N	
Stuttgart-Mühlhausen	506(11)	Western	Adult	Male	N	N		N	
Stuttgart-Mühlhausen	508(14)	Western	Adult	Female	N	N		N	
Stuttgart-Mühlhausen	510(19)	Western	18-20y	Male	N	N		N	
Stuttgart-Mühlhausen	511(23)	Western	45-50y	Female	N	**Y**	Healed	N	
Stuttgart-Mühlhausen	512(28)	Western	12-14y	Female	N	N		N	
Stuttgart-Mühlhausen	513(29)	Western	7-10y	Female	N	**Y**	Healed	N	
Stuttgart-Mühlhausen	514(30)	Western	3-5y	Male	N	N		**Y**	Healed
Stuttgart-Mühlhausen	515(31)	Western	46-59y	Female	N	N		N	
Stuttgart-Mühlhausen	516(33)	Western	35-40y	Female	N	**Y**	Healed	N	
Stuttgart-Mühlhausen	517(34)	Western	25-30y	Female	N	**Y**	Healed	N	
Stuttgart-Mühlhausen	518(38)	Western	40-45y	Male	N	**Y**	Healed	N	
Stuttgart-Mühlhausen	519(39)	Western	35-40y	Male	N	N		N	
Stuttgart-Mühlhausen	520(41)	Western	12-14y	Female	N	**Y**	Healed	**Y**	Active
Stuttgart-Mühlhausen	521(42)	Western	7-9y	Male	N	**Y**	Healed	**Y**	Active
Stuttgart-Mühlhausen	522(43)	Western	30-35y	Male	N	**Y**	Healed	N	
Stuttgart-Mühlhausen	523(46)	Western	10-12y	Female	N	**Y**	Healed	**Y**	Active
Stuttgart-Mühlhausen	524(50)	Western	30-35y	Male	N	**Y**	Healed	N	
Stuttgart-Mühlhausen	525(51)	Western	Adult	Male	N	N		N	
Stuttgart-Mühlhausen	527(55)	Western	30-35y	Female	N	N		N	
Stuttgart-Mühlhausen	528(56)	Western	35-40y	Male	N	**Y**	Healed	N	
Stuttgart-Mühlhausen	529(58)	Western	40-45y	Female	N	N		N	
Stuttgart-Mühlhausen	530(59)	Western	35-40y	Female	**Y**	**Y**	Healed	N	
Stuttgart-Mühlhausen	532(62)	Western	7-10y	Male	N	**Y**	Healed	N	
Stuttgart-Mühlhausen	533(63)	Western	3-4y	Female	N	**Y**	Healed	**Y**	Active
Stuttgart-Mühlhausen	534(69)	Western	35-40y	Female	N	N		N	
Stuttgart-Mühlhausen	535(71)	Western	35-40y	Male	N	**Y**	Healed	N	
Stuttgart-Mühlhausen	536(74)	Western	35-40y	Female	N	N		N	
Stuttgart-Mühlhausen	537(75)	Western	18-20y	Male	N	**Y**	Healed	N	
Stuttgart-Mühlhausen	624(?)	Western	Juvenile	?	N	N		N	
Stuttgart-Mühlhausen	704(7)	Western	12-14y	Male	**Y**	**Y**	Healed	**Y**	Healed
Stuttgart-Mühlhausen	705(?)	Western	30-35y	?	N	N		N	
Schwetzingen	1(1i)	Western	7-13y	?	N	**Y**	Healed	**Y**	Healed
Schwetzingen	1(1ii)	Western	6-7y	?	N	N		N	
Schwetzingen	100(90)	Western	1-2y	Male	N	N		N	

Schwetzingen	105(89)	Western	30-35y	Male	N	**Y**	Healed	**Y**	Active
Schwetzingen	109(96)	Western	12-14y	Male	**Y**	N		N	
Schwetzingen	110(97)	Western	35-40y	Male	N	**Y**	Healed	N	
Schwetzingen	112.1(98)	Western	3-4y	Male	N	N		**Y**	Active
Schwetzingen	113.1(95)	Western	1-2y	Female	N	N		N	
Schwetzingen	114(100)	Western	35-40y	Female	N	**Y**	Healed	N	
Schwetzingen	116.1(102)	Western	6-7y	?	N	N		N	
Schwetzingen	117.1(103)	Western	8-10y	?	N	N		N	
Schwetzingen	120(120)	Western	25-30y	Female	N	N		**Y**	Active
Schwetzingen	122.1(108)	Western	2-3y	?	N	N		**Y**	Healed
Schwetzingen	125.1(106)	Western	40-45y	Male	N	N		N	
Schwetzingen	126.1(107)	Western	30-35y	Male	N	**Y**	Healed	**Y**	Active
Schwetzingen	130(118)	Western	Adult	Male	N	N		N	
Schwetzingen	134.3(117)	Western	14-16y	?	N	N		N	
Schwetzingen	135(119)	Western	30-35y	Female	**Y**	**Y**	Healed	N	
Schwetzingen	144.1(129)	Western	6-7y	Female	N	N		N	
Schwetzingen	145(128)	Western	45-50y	Male	N	N		N	
Schwetzingen	146.1(131)	Western	25-30y	Female	N	N		**Y**	Healed
Schwetzingen	149.1(133)	Western	20-25y	Male	N	**Y**	Healed	**Y**	Healed
Schwetzingen	150.1(130)	Western	46-59y	Male	N	**Y**	Healed	N	
Schwetzingen	155.1(139)	Western	40-45y	Male	**Y**	**Y**	Healed	N	
Schwetzingen	156(140)	Western	Adult	Female	N	N		N	
Schwetzingen	159.1(141)	Western	30-35y	Male	**Y**	N		N	
Schwetzingen	160.1(142)	Western	45-50y	Male	N	**Y**	Healed	**Y**	Active
Schwetzingen	163.1(146)	Western	30-35y	Female	**Y**	N		N	
Schwetzingen	167.1(151)	Western	30-35y	Female	N	N		N	
Schwetzingen	169.1(153)	Western	20-25y	Female	N	N		N	
Schwetzingen	170.1(152)	Western	50-59y	Male	N	**Y**	Healed	N	
Schwetzingen	171.1(154)	Western	40-45y	Male	**Y**	N		N	
Schwetzingen	172.1(155)	Western	12-13y	Male	N	**Y**	Active	**Y**	Active
Schwetzingen	173(156)	Western	4-6y	?	N	N		N	
Schwetzingen	174.1(158)	Western	7-10y	?	N	N		N	
Schwetzingen	177(160)	Western	Adult	?	N	N		N	
Schwetzingen	179(162)	Western	25-35y	?	N	N		N	
Schwetzingen	181(163)	Western	30-35y	Male	N	N		**Y**	Active
Schwetzingen	186.1(169)	Western	30-35y	Male	**Y**	**Y**	Healed	**Y**	Healed
Schwetzingen	187.1(170)	Western	20-25y	Male	**Y**	**Y**	Healed	**Y**	Healed
Schwetzingen	189(168)	Western	45-50y	Male	N	**Y**	Healed	**Y**	Healed
Schwetzingen	194.1(176)	Western	25-30y	Female	**Y**	**Y**	Healed	**Y**	Healed
Schwetzingen	198.1(177)	Western	35-40y	Male	**Y**	**Y**	Healed	**Y**	Active
Schwetzingen	199(179)	Western	18-20y	Female	**Y**	N		N	
Schwetzingen	202.1(182)	Western	7-13y	?	N	N		N	
Schwetzingen	205.1(184)	Western	40-45y	Female	N	**Y**	Healed	**Y**	Healed
Schwetzingen	206(186)	Western	Adult	?	N	N		N	
Schwetzingen	207.1(187)	Western	35-40y	Male	N	N		N	
Schwetzingen	209.1(189)	Western	16-18y	Male	**Y**	**Y**	Healed	**Y**	Active
Schwetzingen	210.1(191)	Western	3-4y	Female	N	N		**Y**	Healed
Schwetzingen	211.1(190)	Western	46-55y	Female	N	N		N	
Schwetzingen	219.1(197)	Western	4-6y	Female	N	N		N	
Schwetzingen	225.1(201)	Western	30-35y	Female	N	**Y**	Active	**Y**	Healed
Schwetzingen	230(207)	Western	Adult	?	N	N		N	
Schwetzingen	232(205)	Western	30-35y	Male	N	N		N	

Schwetzingen	234(208)	Western	Adult	?	N	N		N	
Schwetzingen	236.1(212)	Western	Adult	?	N	N		N	
Schwetzingen	24(13)	Western	1-6y	?	N	N		N	
Schwetzingen	244(215)	Western	12-14y	Female	**Y**	N		N	
Schwetzingen	248(219)	Western	40-45y	Female	N	**Y**	Healed	N	
Schwetzingen	25(12)	Western	14-17y	Male	N	**Y**	Healed	N	
Schwetzingen	26.1(14)	Western	35-40y	Male	N	N		N	
Schwetzingen	28.1(17)	Western	18-25y	Female	N	**Y**	Healed	**Y**	Healed
Schwetzingen	29(22)	Western	4-6y	Female	N	N		N	
Schwetzingen	30.1(16)	Western	35-40y	Male	N	**Y**	Healed	**Y**	Active
Schwetzingen	31(25)	Western	Adult	Female	N	N		N	
Schwetzingen	32(18)	Western	30-35y	Female	**Y**	**Y**	Healed	N	
Schwetzingen	33(30)	Western	45-50y	Female	N	**Y**	Healed	N	
Schwetzingen	35.1(31)	Western	14-17y	Male	N	**Y**	Healed	**Y**	Active
Schwetzingen	37(36)	Western	12-14y	Female	**Y**	**Y**	Healed	**Y**	Healed
Schwetzingen	4(4)	Western	25-30y	Male	**Y**	**Y**	Healed	**Y**	Active
Schwetzingen	46(38)	Western	40-45y	Male	N	**Y**	Healed	N	
Schwetzingen	49(42)	Western	30-35y	Male	N	N		N	
Schwetzingen	54.1(45)	Western	14-17y	?	N	N		N	
Schwetzingen	55.1(35)	Western	35-40y	Male	N	**Y**	Healed	N	
Schwetzingen	57.1(51)	Western	1-2y	Male	N	N		N	
Schwetzingen	58(48)	Western	30-35y	Female	N	N		N	
Schwetzingen	59.1(43)	Western	35-40y	Male	N	N		**Y**	Healed
Schwetzingen	6(5)	Western	40-45y	Male	**Y**	**Y**	Healed	N	
Schwetzingen	60(53)	Western	6-7y	Female	N	N		**Y**	Active
Schwetzingen	61.1(55)	Western	Adult	Female	N	N		N	
Schwetzingen	63(56)	Western	35-40y	Male	N	**Y**	Healed	**Y**	Healed
Schwetzingen	64.1(57)	Western	35-40y	Female	N	**Y**	Healed	N	
Schwetzingen	66.1(61)	Western	Adult	Male	N	N		N	
Schwetzingen	70.1(54)	Western	40-45y	Male	**Y**	**Y**	Healed	**Y**	Healed
Schwetzingen	72.1(65)	Western	40-45y	Female	**Y**	N		**Y**	Healed
Schwetzingen	73.2(46)	Western	40-45y	Female	N	**Y**	Healed	N	
Schwetzingen	74.1(66)	Western	Adult	Male	N	N		N	
Schwetzingen	75.1(63)	Western	7-8y	Female	N	**Y**	Healed	**Y**	Active
Schwetzingen	80(72)	Western	9-10y	?	**Y**	N		N	
Schwetzingen	81.1(67)	Western	40-45y	Female	**Y**	N		N	
Schwetzingen	82.1(71)	Western	35-40y	Male	N	**Y**	Healed	**Y**	Active
Schwetzingen	84.1(75)	Western	9-10y	?	N	N		N	
Schwetzingen	86.1(73)	Western	25-30y	Male	N	N		**Y**	Healed
Schwetzingen	87.1(77)	Western	40-45y	Male	N	N		N	
Schwetzingen	88(76)	Western	5-6y	Female	N	N		N	
Schwetzingen	89.2(78)	Western	Adult	?	N	N		N	
Schwetzingen	90.4(74)	Western	Adult	?	N	N		N	
Schwetzingen	94.1(86)	Western	2-3y	Female	N	N		N	
Schwetzingen	97.1(85)	Western	4-5y	Male	N	N		**Y**	Active
Vedrovice	1/63	Central	1-2yrs	Male	N	N		N	
Vedrovice	1/85	Central	25-30yrs	Male	N	**Y**	Healed	N	
Vedrovice	10/74	Central	45-50yrs	Male	N	N		N	
Vedrovice	10/89	Central	35-45yrs	Female	**Y**	**Y**	Healed	N	
Vedrovice	100/81	Central	30-35yrs	Female	N	N		N	
Vedrovice	101/81	Central	40-45yrs	Male	N	N		N	
Vedrovice	102/81	Central	40-45yrs	Female	N	N		N	

Vedrovice	103/81	Central	30-35yrs	Female	N	N		N	
Vedrovice	104/81	Central	45-59yrs	Female	N	N		N	
Vedrovice	105/81	Central	18-20yrs	Male	**Y**	**Y**	Healed	N	
Vedrovice	106/82	Central	14-17yrs	Male	N	**Y**	Healed	N	
Vedrovice	107/82	Central	18-21yrs	Female	N	N		N	
Vedrovice	108/84	Central	Adult	Male	N	N		N	
Vedrovice	109/84	Central	4-5yrs	Male	N	**Y**	Healed	**Y**	Active
Vedrovice	11/74	Central	25-35yrs	Male	N	N		N	
Vedrovice	11/97	Central	40-45yrs	Female	N	**Y**	Healed	N	
Vedrovice	12/74	Central	Adult	Male	N	N		N	
Vedrovice	12/96	Central	3-4yrs	Male	N	N		N	
Vedrovice	13	Central	6-12m	?	N	N		N	
Vedrovice	13/75	Central	40-45yrs	Male	**Y**	N		N	
Vedrovice	13/97	Central	1.5-2.5yrs	?	N	N		N	
Vedrovice	14/75	Central	20-25yrs	Female	N	N		N	
Vedrovice	14/97	Central	Adult	?	N	**Y**	Healed	N	
Vedrovice	15/75	Central	35-45yrs	Male	N	N		N	
Vedrovice	16/75	Central	2-4yrs	Female	N	N		N	
Vedrovice	17/75	Central	3-4yrs	?	N	N		N	
Vedrovice	18/75	Central	6-7yrs	Male	N	N		N	
Vedrovice	19/75	Central	30-35yrs	Male	N	**Y**	Active	**Y**	Healed
Vedrovice	2/63	Central	5-6yrs	Male	N	**Y**	Healed	N	
Vedrovice	2/85	Central	30-35yrs	Male	N	N		N	
Vedrovice	20/75	Central	3-4yrs	Male	N	N		**Y**	Healed
Vedrovice	21/75	Central	35-45yrs	Female	N	N		N	
Vedrovice	22/75	Central	40-45yrs	Female	**Y**	**Y**	Active	N	
Vedrovice	23/75	Central	18-21yrs	Male	**Y**	N		**Y**	Healed
Vedrovice	24/75	Central	7-12yrs	Female	N	N		N	
Vedrovice	25/75	Central	Adult	Male	N	N		N	
Vedrovice	27/76	Central	Adult	Female	N	N		N	
Vedrovice	28/76	Central	4-5yrs	Male	N	N		N	
Vedrovice	29/76	Central	20-25yrs	Female	**Y**	N		N	
Vedrovice	3/66	Central	8-9yrs	Female	N	**Y**	Healed	N	
Vedrovice	3/86	Central	1-2yrs	?	N	N		**Y**	Active
Vedrovice	30/76	Central	18-25yrs	Female	**Y**	N		N	
Vedrovice	31/76	Central	Adult	?	N	N		N	
Vedrovice	32/76	Central	10-11yrs	Female	N	N		N	
Vedrovice	35/76	Central	Adult	Female	N	N		N	
Vedrovice	36/76	Central	40-45yrs	Female	N	N		N	
Vedrovice	37/76	Central	11-12yrs	?	N	N		N	
Vedrovice	38/76	Central	40-45yrs	Female	N	N		N	
Vedrovice	39/76	Central	2-3yrs	Male	N	N		N	
Vedrovice	4/69	Central	6-7yrs	?	N	N		N	
Vedrovice	40/76	Central	9-10yrs	Female	N	N		N	
Vedrovice	42/77	Central	25-30yrs	Male	N	N		N	
Vedrovice	43/77	Central	18-25yrs	Female	N	**Y**	Healed	N	
Vedrovice	44/77	Central	Adult	?	N	N		N	
Vedrovice	45/77	Central	40-45yrs	Male	N	**Y**	Healed	N	
Vedrovice	46/77	Central	25-30yrs	Male	N	N		N	
Vedrovice	48/77	Central	18-20yrs	Female	N	N		N	
Vedrovice	5	Central	4-5yrs	?	N	N		N	
Vedrovice	5/71	Central	6-8yrs	Female	**Y**	N		N	

Vedrovice	5/88	Central	2-3yrs	Male	N	N		N	
Vedrovice	50/77	Central	Adult	Male	N	N		N	
Vedrovice	51/77	Central	Adult	?	N	N		N	
Vedrovice	54/78	Central	20-25yrs	Male	N	N		N	
Vedrovice	55/78	Central	Adult	?	N	N		N	
Vedrovice	56/78	Central	Adult	?	N	N		N	
Vedrovice	57/78	Central	40-45y	Male	N	N		N	
Vedrovice	59/78	Central	30-35y	?	N	**Y**	Active	N	
Vedrovice	6/72	Central	2-3y	?	N	N		N	
Vedrovice	6/88	Central	46-49y	Female	N	N		N	
Vedrovice	61/78	Central	30-35y	?	N	N		N	
Vedrovice	62/78	Central	30-35y	Male	**Y**	N		N	
Vedrovice	63/78	Central	30-35y	?	N	N		N	
Vedrovice	64/78	Central	18-20y	Female	**Y**	N		N	
Vedrovice	66/78	Central	35-40y	Male	N	**Y**	Active	N	
Vedrovice	67/78	Central	30-35y	?	**Y**	N		N	
Vedrovice	68/78	Central	40-45y	Female	**Y**	N		N	
Vedrovice	69/78	Central	Adult	?	N	N		N	
Vedrovice	7/72	Central	0m	?	N	N		N	
Vedrovice	7/88	Central	35-40y	Female	N	N		N	
Vedrovice	70/79	Central	Adult	?	N	N		N	
Vedrovice	71/79	Central	35-40y	Male	N	N		N	
Vedrovice	72/79	Central	35-40y	Female	N	N		N	
Vedrovice	73/79	Central	25-30y	Male	**Y**	N		N	
Vedrovice	74/79	Central	Adult	Female	N	N		N	
Vedrovice	75/79	Central	30-35y	Female	N	N		N	
Vedrovice	76/79	Central	30-35y	Male	**Y**	N		N	
Vedrovice	77/79	Central	35-40y	Male	N	N		N	
Vedrovice	78/79	Central	6-7y	Female	**Y**	N		**Y**	Active
Vedrovice	79/79	Central	25-30y	Male	N	N		**Y**	Healed
Vedrovice	8/74	Central	37-38wks	Male	N	N		N	
Vedrovice	8/88	Central	11-12y	Female	N	N		N	
Vedrovice	80/79	Central	40-45y	Female	**Y**	N		N	
Vedrovice	81a/79	Central	20-25y	Female	N	N		N	
Vedrovice	81b/79	Central	Juvenile	Female	N	N		N	
Vedrovice	82/79	Central	46-59y	Male	N	N		N	
Vedrovice	83/80	Central	36-45y	Female	N	N		N	
Vedrovice	84/80	Central	7-9y	?	N	N		N	
Vedrovice	86/80	Central	30-35y	Female	**Y**	N		**Y**	Healed
Vedrovice	87/80	Central	Adult	Male	N	N		N	
Vedrovice	88/80	Central	30-35y	Male	**Y**	N		N	
Vedrovice	89/80	Central	Adult	Male	N	N		N	
Vedrovice	9/74	Central	46-59y	Female	N	N		N	
Vedrovice	9/88	Central	20-25y	Female	N	N		N	
Vedrovice	90/80	Central	Adult	?	N	N		N	
Vedrovice	91/80	Central	20-25y	Male	**Y**	**Y**	Healed	N	
Vedrovice	93a/80	Central	18-20y	Female	N	N		**Y**	Healed
Vedrovice	93b/80	Central	birth-1m	?	N	N		N	
Vedrovice	94/80	Central	20-25y	Female	**Y**	N		N	
Vedrovice	95/80	Central	40-45y	?	N	N		N	
Vedrovice	96/80	Central	2-3y	Male	N	N		N	
Vedrovice	97/80	Central	30-35y	Male	N	N		N	

Vedrovice	98/81	Central	Adult	?	N	N		N	
Vedrovice	99/81	Central	25-30y	Male	**Y**	**Y**	Healed	**Y**	Active

Supplementary Table S4: Results of Kruskal-Wallis analysis of variance in age at onset of hypoplasia formation between LBK populations. Much of the total significance was accounted for by inter-site variation in the average age at onset within the adult female and juvenile demographic groupings. For both groupings onset was significantly later in populations from the western LBK than populations from the rest of the geographic distribution. SW - Schwetzingen; SM - Stuttgart-Mühlhausen; V - Vedrovice; HK - Nitra-Horné Krškany; PF - Polgár-Ferenci-hát. n - number of defects; SD - one standard deviation. Statistically significant results are highlighted in bold font.

Variable		n	Mean (years)	SD	H	df	p
**Total Population**	SW	104	3.89	1.09	31.90	4	**0.000**
	SM	86	3.87	0.92			
	V	90	3.69	0.82			
	HK	53	2.94	1.37			
	PF	95	3.23	1.33			
**Adult**	SW	68	3.96	1.03	8.63	4	0.071
	SM	72	3.80	0.90			
	V	91	3.73	0.80			
	HK	33	3.58	0.99			
	PF	35	4.16	0.97			
**Juvenile**	SW	36	3.75	1.20	35.53	4	**0.000**
	SM	14	4.23	0.95			
	V	4	2.77	0.69			
	HK	20	1.89	1.26			
	PF	60	2.68	1.21			
**Male**	SW	40	3.65	0.80	9.01	4	0.061
	SM	50	3.89	0.80			
	V	45	3.81	0.85			
	HK	23	3.72	1.12			
	PF	14	4.36	0.56			
**Female**	SW	28	4.40	1.18	12.50	4	**0.014**
	SM	15	3.70	1.18			
	V	40	3.62	0.77			
	HK	10	3.24	0.50			
	PF	21	4.02	1.16			

Supplementary Table S5: Results of unpaired Wilcoxon rank sum tests to assess differences in age at onset of hypoplasia formation between adults (A) and juveniles (J), and also between adult males (M) and females (F) within LBK populations. Differences in age at onset between males and females was only significant at Schwetzingen, within the western LBK, but adult and juvenile onset differed significantly in all populations from the central and eastern LBK. n - number of defects; SD - one standard deviation. Statistically significant results are highlighted in bold font.

Variable		n	Mean (years)	SD	W	p
**Schwetzingen**	***A***	68	3.96	1.03	1363	0.344
	J	36	3.75	1.20		
	M	40	3.65	0.80	340	**0.006**
	F	28	4.40	1.18		
**Stuttgart-Mühlhausen**	***A***	72	3.80	0.90	345	0.064
	J	14	4.23	0.95		
	M	50	3.89	0.80	427	0.423
	F	15	3.70	1.18		
**Vedrovice**	***A***	91	3.73	0.80	301	**0.028**
	J	4	2.77	0.69		
	M	45	3.81	0.85	1023.5	0.279
	F	40	3.62	0.77		
**Nitra Horné Krškany**	***A***	33	3.58	0.99	549	**0.000**
	J	20	1.89	1.26		
	M	23	3.72	1.12	143	0.287
	F	10	3.24	0.50		
**Polgár-Ferenci-hát**	***A***	35	4.16	0.97	1736	**0.000**
	J	60	2.68	1.21		
	M	14	4.36	0.56	172	0.414
	F	21	4.02	1.16		

Supplementary Table S6: Isotopic data for human remains used to plot Fig.4. Specific location of bone tested is included to illustrate where inter-study results are comparable. Long - unspecified long bone; MC - metacarpal; MT - metatarsal; OC - *os coxae*; ns - not specified; ns* - not specified but used in study suggesting compatibility with collagen extracted from ribs.

Site	Country	Burial	Sex	$\delta^{13}C$	$\delta^{15}N$	Bone	Source
Aiterhofen	Germany	9	Female	-20.5	10.2	ns	6
Aiterhofen	Germany	10	Male	-20.2	10.7	ns	6
Aiterhofen	Germany	12	Male	-20.4	10.4	ns	6
Aiterhofen	Germany	13	Male	-20.4	9.7	ns	6
Aiterhofen	Germany	14	Female	-20.5	9.9	ns	6
Aiterhofen	Germany	15	Male	-20.1	10.7	ns	6
Aiterhofen	Germany	18	Male	-20.1	10.2	ns	6
Aiterhofen	Germany	19A	Female	-20.4	9.6	ns	6
Aiterhofen	Germany	21	Male	-20.1	9	ns	6
Aiterhofen	Germany	23A	Juvenile	-20.5	10	ns	6
Aiterhofen	Germany	24A	Male	-20.6	9.8	ns	6
Aiterhofen	Germany	25	Male	-20.1	9.6	ns	6
Aiterhofen	Germany	26A	Male	-20.2	9.7	ns	6
Aiterhofen	Germany	28	Male	-20.3	10	ns	6
Aiterhofen	Germany	31	Male	-20.3	9.5	ns	6
Aiterhofen	Germany	41	Juvenile	-20.7	9.2	ns	6
Aiterhofen	Germany	42	Male	-20	10	ns	6
Aiterhofen	Germany	43	Male	-20.8	9.1	ns	6
Aiterhofen	Germany	47	Juvenile	-20.4	9.4	ns	6
Aiterhofen	Germany	48	Male	-20.7	9.9	ns	6
Aiterhofen	Germany	55	Female	-20.2	9.9	ns	6
Aiterhofen	Germany	56	Male	-20.3	10.2	ns	6
Aiterhofen	Germany	57	Male	-20.2	9.7	ns	6
Aiterhofen	Germany	58	Juvenile	-20.4	11.1	ns	6
Aiterhofen	Germany	60	Female	-20.5	9.9	ns	6
Aiterhofen	Germany	68	Female	-20.4	9.9	ns	6
Aiterhofen	Germany	69	Female	-20.5	9.5	ns	6
Aiterhofen	Germany	74	Male	-20.6	9.4	ns	6
Aiterhofen	Germany	78	Male	-20.5	9.8	ns	6
Aiterhofen	Germany	85	Male	-20.5	10.2	ns	6
Aiterhofen	Germany	88	Juvenile	-20.7	10.1	ns	6
Aiterhofen	Germany	89	Female	-20.1	10.1	ns	6
Aiterhofen	Germany	91	Female	-20.3	9.1	ns	6
Aiterhofen	Germany	94	Male	-20.7	9.6	ns	6
Aiterhofen	Germany	99	Female	-20.6	9.7	ns	6
Aiterhofen	Germany	100	Female	-20.8	9.5	ns	6
Aiterhofen	Germany	102	Male	-20.5	10.5	ns	6
Aiterhofen	Germany	106	Female	-20.1	9	ns	6
Aiterhofen	Germany	108	Male	-20.3	10	ns	6
Aiterhofen	Germany	109	Female	-20.2	10.1	ns	6
Aiterhofen	Germany	111	Female	-20.4	9.8	ns	6
Aiterhofen	Germany	115A	Male	-20.4	9.8	ns	6
Aiterhofen	Germany	116A	Female	-20.5	9.9	ns	6
Aiterhofen	Germany	118	Male	-20.7	9.8	ns	6
Aiterhofen	Germany	119	Male	-21	9.5	ns	6

Aiterhofen	Germany	130	Male	-20.9	9.5	ns	6
Aiterhofen	Germany	137	Female	-20.5	10.2	ns	6
Aiterhofen	Germany	139A	Female	-20.6	9.4	ns	6
Aiterhofen	Germany	140	Female	-20.3	9.7	ns	6
Aiterhofen	Germany	141	Male	-20	10.3	ns	6
Aiterhofen	Germany	142	Male	-20.6	9.2	ns	6
Aiterhofen	Germany	144	Female	-20.7	9.5	ns	6
Aiterhofen	Germany	147	Juvenile	-20.6	9.8	ns	6
Aiterhofen	Germany	148	Juvenile	-20.6	9.4	ns	6
Aiterhofen	Germany	150	Female	-20.1	9.8	ns	6
Aiterhofen	Germany	158	Female	-21	10	ns	6
Asparn an der Zaya/Schletz	Austria	281	Male	-19.7	9.4	ns	2
Asparn an der Zaya/Schletz	Austria	685	Male	-19.6	10.2	ns	2
Asparn an der Zaya/Schletz	Austria	573	Male	-19.6	9.4	ns	2
Asparn an der Zaya/Schletz	Austria	119,79A	Male	-19.6	9	ns	2
Asparn an der Zaya/Schletz	Austria	75A,77	Male	-19.5	10.4	ns	2
Asparn an der Zaya/Schletz	Austria	649	Juvenile	-19.5	9.4	ns	2
Asparn an der Zaya/Schletz	Austria	4518	Male	-19.5	9.4	ns	2
Asparn an der Zaya/Schletz	Austria	2873	Juvenile	-20	10.1	ns	2
Asparn an der Zaya/Schletz	Austria	558572	Juvenile	-20.3	8.9	ns	2
Asparn an der Zaya/Schletz	Austria	5839	Male	-19.6	9.4	ns	2
Asparn an der Zaya/Schletz	Austria	601	Juvenile	-19.6	9.1	ns	2
Asparn an der Zaya/Schletz	Austria	345	Juvenile	-19.9	9.1	ns	2
Asparn an der Zaya/Schletz	Austria	655	Juvenile	-19.5	9.7	ns	2
Asparn an der Zaya/Schletz	Austria	285	Juvenile	-18.8	12.9	ns	2
Asparn an der Zaya/Schletz	Austria	1996 7	Juvenile	-19.7	8.7	ns	2
Asparn an der Zaya/Schletz	Austria	2490	Female	-19.4	8.8	ns	2
Asparn an der Zaya/Schletz	Austria	360	Male	-19.7	10	ns	2
Asparn an der Zaya/Schletz	Austria	2526	Male	-19.9	10.4	ns	2
Balatonszarszo-Kis-erdei-dulo	Hungary	278	Juvenile	-20	8.9	Long	5
Balatonszarszo-Kis-erdei-dulo	Hungary	288	Juvenile	-19.9	10.2	Long	5
Balatonszarszo-Kis-erdei-dulo	Hungary	289	Juvenile	-20.1	9.8	Long	5
Balatonszarszo-Kis-erdei-dulo	Hungary	510	Male	-20	8.8	MC	5
Balatonszarszo-Kis-erdei-dulo	Hungary	531	Male	-19.9	9.5	MC	5
Balatonszarszo-Kis-erdei-dulo	Hungary	554	Female	-19.7	9.3	Long	5
Balatonszarszo-Kis-erdei-dulo	Hungary	711	Juvenile	-19.1	9.5	Long	5
Balatonszarszo-Kis-erdei-dulo	Hungary	712	Male	-19.9	9.3	Long	5
Balatonszarszo-Kis-erdei-dulo	Hungary	766	Male	-20	9.9	Long	5
Balatonszarszo-Kis-erdei-dulo	Hungary	767	Male	-19.9	10.2	Long	5
Balatonszarszo-Kis-erdei-dulo	Hungary	768	Juvenile	-20.2	9.8	Long	5
Balatonszarszo-Kis-erdei-dulo	Hungary	769	Juvenile	-20.2	9.8	Long	5
Balatonszarszo-Kis-erdei-dulo	Hungary	770	Male	-20	9.5	MC	5
Balatonszarszo-Kis-erdei-dulo	Hungary	771	Male	-19.7	8.9	MC	5
Balatonszarszo-Kis-erdei-dulo	Hungary	772	Juvenile	-19.6	9.6	Long	5
Balatonszarszo-Kis-erdei-dulo	Hungary	773	Female	-20	8.4	MC	5
Balatonszarszo-Kis-erdei-dulo	Hungary	774	Female	-20.1	8.5	MC	5
Balatonszarszo-Kis-erdei-dulo	Hungary	775	Juvenile	-19.6	9.1	Scapula	5
Balatonszarszo-Kis-erdei-dulo	Hungary	776	Female	-20	10.1	Long	5
Balatonszarszo-Kis-erdei-dulo	Hungary	777	Female	-19.9	9.2	Long	5
Balatonszarszo-Kis-erdei-dulo	Hungary	778	Female	-20.5	9.6	Long	5
Balatonszarszo-Kis-erdei-dulo	Hungary	779	Juvenile	-19.3	11.4	OC	5
Balatonszarszo-Kis-erdei-dulo	Hungary	780	Male	-19.7	9.3	Phalanx	5

Balatonszarszo-Kis-erdei-dulo	Hungary	781	Male	-19.9	9.4	Long	5
Balatonszarszo-Kis-erdei-dulo	Hungary	782	Female	-19.8	9.4	MC	5
Balatonszarszo-Kis-erdei-dulo	Hungary	783	Female	-19.9	9.75	Long	5
Balatonszarszo-Kis-erdei-dulo	Hungary	784	Male	-20.3	10.1	Long	5
Balatonszarszo-Kis-erdei-dulo	Hungary	785	Male	-20.1	9.6	Long	5
Balatonszarszo-Kis-erdei-dulo	Hungary	786	Female	-20.2	9.6	Long	5
Balatonszarszo-Kis-erdei-dulo	Hungary	787	Female	-19.4	9.2	MC	5
Balatonszarszo-Kis-erdei-dulo	Hungary	789	Female	-20	9.7	Long	5
Balatonszarszo-Kis-erdei-dulo	Hungary	790	Juvenile	-19.3	10.7	Long	5
Balatonszarszo-Kis-erdei-dulo	Hungary	791	Female	-19.9	8.1	MC	5
Balatonszarszo-Kis-erdei-dulo	Hungary	792	Male	-19.9	9.5	MC	5
Balatonszarszo-Kis-erdei-dulo	Hungary	793	Male	-19.7	9.7	MC	5
Balatonszarszo-Kis-erdei-dulo	Hungary	794	Female	-19.8	10.1	Rib	5
Balatonszarszo-Kis-erdei-dulo	Hungary	795	Female	-19.8	9.8	Cranium	5
Balatonszarszo-Kis-erdei-dulo	Hungary	796	Juvenile	-19.6	9.6	Patella	5
Balatonszarszo-Kis-erdei-dulo	Hungary	797	Juvenile	-19.9	9.3	Long	5
Balatonszarszo-Kis-erdei-dulo	Hungary	798	Female	-20.1	9.8	Long	5
Balatonszarszo-Kis-erdei-dulo	Hungary	799	Male	-20.3	10	Long	5
Brno-Stary Liskovec/Novy Liskovec	Czech Republic	2565/802	Male	-19.6	9.9	Rib	3
Brno-Stary Liskovec/Novy Liskovec	Czech Republic	5817/803	Male	-19.7	10.3	Rib	3
Brno-Stary Liskovec/Novy Liskovec	Czech Republic	7714/805	Male	-19.9	9.8	Rib	3
Brno-Stary Liskovec/Novy Liskovec	Czech Republic	7727/806	Male	-19.5	10.4	Rib	3
Derenburg	Germany	18	Juvenile	-20.5	8.6	Cranium	7
Derenburg	Germany	20	Juvenile	-20	8.7	Cranium	7
Derenburg	Germany	23	Juvenile	-19.8	8.9	Rib	7
Derenburg	Germany	24	Juvenile	-19.1	11.2	Rib	7
Derenburg	Germany	26	Juvenile	-19.2	9.7	Cranium	7
Derenburg	Germany	27	Juvenile	-19.5	9.5	Long	7
Derenburg	Germany	47	Juvenile	-19	11.5	Cranium	7
Derenburg	Germany	19	Juvenile	-19.6	8.7	Rib	7
Derenburg	Germany	29	Juvenile	-19.6	8.6	Rib	7
Derenburg	Germany	33	Juvenile	-19.4	8.8	Rib	7
Derenburg	Germany	37	Juvenile	-20	9	Rib	7
Derenburg	Germany	17	Male	-19.4	9.2	Rib	7
Derenburg	Germany	31	Male	-19.7	8.3	Rib	7
Derenburg	Germany	34	Male	-19.7	9.2	Rib	7
Derenburg	Germany	28	Male	-19.2	9.2	Rib	7
Derenburg	Germany	42	Male	-19.8	8.5	Rib	7
Derenburg	Germany	46	Male	-19.4	8.6	Rib	7
Derenburg	Germany	12	Male	-20.2	8.3	Rib	7
Derenburg	Germany	43	Juvenile	-19.7	8.3	Rib	7
Derenburg	Germany	9	Female	-20.2	8.4	Rib	7
Derenburg	Germany	21	Female	-19.8	8.5	Rib	7
Derenburg	Germany	30	Female	-19.4	9.2	Rib	7
Derenburg	Germany	38	Female	-20.2	10.1	Femur	7
Derenburg	Germany	48	Female	-19.3	9.1	Rib	7
Derenburg	Germany	49	Female	-19.6	9.2	Rib	7
Derenburg	Germany	32	Female	-19.4	8.6	Rib	7
Derenburg	Germany	44	Female	-19.8	8.4	Rib	7
Derenburg	Germany	45	Female	-19.8	8.2	Rib	7
Derenburg	Germany	16	Female	-20.6	8.6	Long	7
Derenburg	Germany	35	Female	-20	8.4	Rib	7

Derenburg	Germany	40	Female	-20.1	8.8	Rib	7
Derenburg	Germany	41	Female	-19.9	8.9	Long	7
Derenburg	Germany	22	Female	-20.2	7.9	Long	7
Derenburg	Germany	47	Female	-20	8.7	Long	7
Derenburg	Germany	39	Female	-20.2	8.6	Rib	7
Ensisheim les Octrois	France	1	Female	-20.5	8.8	Rib	1
Ensisheim les Octrois	France	2	Female	-20.3	9.9	Rib	1
Ensisheim les Octrois	France	3	Male	-20.2	9.9	Rib	1
Ensisheim les Octrois	France	4	Female	-20.2	9.1	Rib	1
Ensisheim les Octrois	France	5	Juvenile	-20.4	8.7	Rib	1
Ensisheim les Octrois	France	7	Female	-20.1	8.8	Rib	1
Ensisheim les Octrois	France	8	Male	-20.1	9.4	Rib	1
Ensisheim les Octrois	France	10	Female	-20.1	9.1	Rib	1
Ensisheim les Octrois	France	11	Male	-20.1	8.9	Rib	1
Ensisheim les Octrois	France	12	Male	-20.1	9	Rib	1
Ensisheim les Octrois	France	14	Juvenile	-20	9.3	Rib	1
Ensisheim les Octrois	France	15	Male	-20	9.7	Rib	1
Ensisheim les Octrois	France	16	Female	-20.5	8.8	Rib	1
Ensisheim les Octrois	France	18	Juvenile	-20.3	9.2	Rib	1
Ensisheim les Octrois	France	19	Male	-20	8.8	Rib	1
Ensisheim les Octrois	France	20	Male	-19.8	9.9	Rib	1
Ensisheim les Octrois	France	21	Male	-20.1	9.7	Rib	1
Ensisheim les Octrois	France	22	Male	-19.9	10	Rib	1
Ensisheim les Octrois	France	23	Juvenile	-20.1	9	Rib	1
Ensisheim les Octrois	France	24	Female	-20	9.5	Rib	1
Ensisheim les Octrois	France	25	Juvenile	-20.2	9.6	Tibia	1
Ensisheim les Octrois	France	26	Female	-20.3	9.3	Rib	1
Ensisheim les Octrois	France	27	Female	-20.3	9.1	Rib	1
Ensisheim les Octrois	France	28	Female	-20.3	8.9	Rib	1
Ensisheim les Octrois	France	29	Female	-20.4	9.4	Rib	1
Ensisheim les Octrois	France	30	Male	-19.9	8.8	Rib	1
Ensisheim les Octrois	France	31	Juvenile	-19.7	9.8	Rib	1
Ensisheim les Octrois	France	32	Male	-20.2	9.6	Rib	1
Ensisheim les Octrois	France	33	Male	-20.2	8.4	Rib	1
Ensisheim les Octrois	France	34	Male	-20.4	9.5	Rib	1
Ensisheim les Octrois	France	35	Male	-20.2	9.6	Rib	1
Ensisheim les Octrois	France	36	Male	-20.6	9.2	Rib	1
Ensisheim les Octrois	France	37	Male	-20	9.8	Rib	1
Ensisheim les Octrois	France	39	Female	-19.8	9.4	Rib	1
Ensisheim les Octrois	France	40	Male	-20.1	10.9	Rib	1
Ensisheim les Octrois	France	41	Female	-20.3	8.7	Rib	1
Ensisheim les Octrois	France	42	Female	-20.4	8.9	Rib	1
Ensisheim les Octrois	France	44	Male	-20	9.4	Rib	1
Ensisheim les Octrois	France	45	Female	-20.4	9.9	MT	1
Fuzesabony-Gubakut	Hungary	1	Female	-19.9	9.9	Long	5
Fuzesabony-Gubakut	Hungary	2	Juvenile	-20	10.6	Long	5
Fuzesabony-Gubakut	Hungary	3	Female	-19.8	10.5	Long	5
Fuzesabony-Gubakut	Hungary	4	Female	-19.8	10.2	Rib	5
Fuzesabony-Gubakut	Hungary	5	Male	-20.3	10.5	Rib	5
Fuzesabony-Gubakut	Hungary	6	Juvenile	-20.5	10	Cranium	5
Fuzesabony-Gubakut	Hungary	9	Male	-19.9	11.1	Long	5
Fuzesabony-Gubakut	Hungary	10	Male	-19.8	10.5	Rib	5

Fuzesabony-Gubakut	Hungary	11	Juvenile	-20	9.8	Long	5
Fuzesabony-Gubakut	Hungary	13	Juvenile	-19.6	13	Cranium	5
Halberstadt	Germany	8	Juvenile	-19	10.3	Rib	7
Halberstadt	Germany	11.2	Juvenile	-19.5	8.7	Rib	7
Halberstadt	Germany	17.1	Juvenile	-19.6	10.2	Rib	7
Halberstadt	Germany	24	Juvenile	-19.8	8.6	Rib	7
Halberstadt	Germany	29	Juvenile	-19.4	10.5	Rib	7
Halberstadt	Germany	32	Juvenile	-19.5	10	Long	7
Halberstadt	Germany	33	Juvenile	-19.7	8.9	Rib	7
Halberstadt	Germany	37	Juvenile	-20	9	Long	7
Halberstadt	Germany	38	Juvenile	-19.8	7.7	Rib	7
Halberstadt	Germany	7	Juvenile	-19.5	9.1	Rib	7
Halberstadt	Germany	10.1	Juvenile	-19.2	9.9	Rib	7
Halberstadt	Germany	23	Juvenile	-19.7	7.5	Rib	7
Halberstadt	Germany	30	Juvenile	-19.8	7.4	Rib	7
Halberstadt	Germany	31	Juvenile	-19.9	7.7	Cranium	7
Halberstadt	Germany	40	Juvenile	-18.5	9.9	Rib	7
Halberstadt	Germany	9	Juvenile	-20	10.5	Rib	7
Halberstadt	Germany	28	Male	-19.4	9.2	Rib	7
Halberstadt	Germany	35	Male	-19.7	8.7	Rib	7
Halberstadt	Germany	15	Male	-19.8	8.4	Rib	7
Halberstadt	Germany	2	Male	-20.1	8	Rib	7
Halberstadt	Germany	19.1	Male	-19.6	11.7	Rib	7
Halberstadt	Germany	27	Male	-19.6	9	Rib	7
Halberstadt	Germany	16	Juvenile	-19.8	8.3	Rib	7
Halberstadt	Germany	41	Juvenile	-19.6	8.1	Rib	7
Halberstadt	Germany	18	Male	-20.1	8	Rib	7
Halberstadt	Germany	1	Female	-19.1	9.1	Rib	7
Halberstadt	Germany	17	Female	-19.5	8.6	Rib	7
Halberstadt	Germany	34	Female	-20.2	8.7	Rib	7
Halberstadt	Germany	36	Female	-19.9	8	Rib	7
Halberstadt	Germany	25	Female	-19.6	8.2	Rib	7
Halberstadt	Germany	42	Female	-19.7	8.2	Rib	7
Halberstadt	Germany	22	Female	-19.9	8.3	Rib	7
Halberstadt	Germany	26	Female	-20.2	8.6	Rib	7
Halberstadt	Germany	39	Female	-19.7	8.4	Rib	7
Halberstadt	Germany	20	Female	-20.1	7.4	Long	7
Herxheim	Germany	HX1	Male	-19.8	9.6	ns	8
Herxheim	Germany	HX2	Female	-19.9	9	ns	8
Herxheim	Germany	HX3	Male	-20.4	9.8	ns	8
Herxheim	Germany	HX4	Male	-20.1	10.1	ns	8
Herxheim	Germany	HX5	Male	-20.2	8.3	ns	8
Herxheim	Germany	HX6	Male	-19.8	10.3	ns	8
Herxheim	Germany	HX8	Male	-20.3	12.1	ns	8
Herxheim	Germany	HX9	Male	-19.7	10.4	ns	8
Herxheim	Germany	HX10	Male	-20.4	9.1	ns	8
Herxheim	Germany	HX11	Male	-20	7.8	ns	8
Herxheim	Germany	HX12	Female	-20.5	8.8	ns	8
Herxheim	Germany	HX13	Female	-20.3	10.2	ns	8
Herxheim	Germany	HX15	Female	-20	9.6	ns	8
Herxheim	Germany	HX17	Female	-20	9.7	ns	8
Herxheim	Germany	HX18	Male	-19.8	10.3	ns	8

Herxheim	Germany	HX19	Female	-19.6	11.5	ns	8
Herxheim	Germany	HX20	Female	-20	8.9	ns	8
Herxheim	Germany	HX21	Male	-20.1	11.3	ns	8
Herxheim	Germany	HX22	Female	-19.8	10.7	ns	8
Jechtingen	Germany	1	Male	-20.4	8.6	Rib	9
Jechtingen	Germany	2	-	-20.7	8.2	Rib	9
Jechtingen	Germany	3	Juvenile	-20.3	9.1	Rib	9
Jechtingen	Germany	5	-	-20.4	9.1	Rib	9
Jechtingen	Germany	6	-	-20.5	8.5	Femur	9
Jechtingen	Germany	7	-	-20.1	9.1	Rib	9
Jechtingen	Germany	8	Female	-20.7	9.6	Rib	9
Jechtingen	Germany	9	-	-20.7	9.9	Rib	9
Jechtingen	Germany	10	-	-20.4	10.6	Cranium	9
Jechtingen	Germany	11	Juvenile	-20.5	9.6	Humerus	9
Jechtingen	Germany	13	-	-20.1	10.8	Clavicle	9
Jechtingen	Germany	14	Female	-20.1	10.6	Rib	9
Jechtingen	Germany	15	Male	-20.2	11.9	Humerus	9
Jechtingen	Germany	16	-	-20.8	10.3	Fibula	9
Jechtingen	Germany	19	Juvenile	-20.5	10.5	Cranium	9
Jechtingen	Germany	20	Juvenile	-20.6	9.1	Ulna	9
Jechtingen	Germany	21	-	-20.6	10.1	Humerus	9
Jechtingen	Germany	22	Female	-20.4	9.9	Rib	9
Jechtingen	Germany	23	-	-20.6	9.6	Humerus	9
Jechtingen	Germany	25	-	-20.7	9.9	Rib	9
Jechtingen	Germany	26	Male	-20.2	10.5	Rib	9
Jechtingen	Germany	27	-	-20.6	10.8	Rib	9
Jechtingen	Germany	28	-	-20.3	11	Fibula	9
Jechtingen	Germany	29	Male	-20.3	9.5	Tibia	9
Jechtingen	Germany	30	-	-20.8	10.2	Femur	9
Jechtingen	Germany	31	Juvenile	-20.7	9	Humerus	9
Jechtingen	Germany	32	Female	-20.7	7.9	Radius	9
Jechtingen	Germany	33	-	-20.6	8.7	Cranium	9
Jechtingen	Germany	34	Female	-20.6	9.7	Cranium	9
Jechtingen	Germany	35	-	-20.9	10	Femur	9
Jechtingen	Germany	36	-	-20.5	10.5	Tibia	9
Jechtingen	Germany	38	Male	-20.7	8.7	Femur	9
Jechtingen	Germany	39	Female	-20.4	9	Phalanx	9
Jechtingen	Germany	40	-	-20.7	8.8	Humerus	9
Jechtingen	Germany	41	Juvenile	-20.6	9.6	Ulna	9
Jechtingen	Germany	42	Female	-20.5	8.9	Rib	9
Jechtingen	Germany	43	Male	-20.2	9.1	Rib	9
Jechtingen	Germany	44A	Female	-20.6	8.4	Rib	9
Jechtingen	Germany	45	-	-20.7	9.3	Femur	9
Jechtingen	Germany	46	Female	-20.9	8.7	Cranium	9
Jechtingen	Germany	47	Juvenile	-20.7	8.6	Cranium	9
Jechtingen	Germany	48	-	-20.6	9.2	Femur	9
Jechtingen	Germany	50	Female	-20.7	8.6	Humerus	9
Jechtingen	Germany	51	Female	-20.7	9.1	Cranium	9
Jechtingen	Germany	52	-	-20.9	9.5	Humerus	9
Jechtingen	Germany	53	Juvenile	-20.8	9.3	Rib	9
Jechtingen	Germany	54	Female	-20.6	9.2	Rib	9
Jechtingen	Germany	55	-	-20.9	8.6	Humerus	9

Jechtingen	Germany	57	Female	-21.2	9.4	Femur	9
Jechtingen	Germany	58	Female	-21.2	9.8	OC	9
Jechtingen	Germany	59	Male	-21.3	10	Rib	9
Jechtingen	Germany	60	Male	-20.4	9.2	Rib	9
Jechtingen	Germany	65	-	-20.6	9.4	Humerus	9
Jechtingen	Germany	68	-	-21.1	8.7	Cranium	9
Jechtingen	Germany	72	Male	-20.7	9.3	Rib	9
Jechtingen	Germany	73	-	-20.6	8.9	Rib	9
Jechtingen	Germany	74	Male	-20.6	9.1	Rib	9
Jechtingen	Germany	75	Female	-20.6	8.6	Rib	9
Jechtingen	Germany	76	Male	-20.5	8.9	Cranium	9
Jechtingen	Germany	77	Male	-20.6	9.1	Rib	9
Jechtingen	Germany	82	Female	-20.4	8.8	Rib	9
Jechtingen	Germany	83	-	-20.8	9	Fibula	9
Jechtingen	Germany	86(87A)	-	-20.7	9.3	Cranium	9
Jechtingen	Germany	88	Male	-20.3	8.9	Humerus	9
Jechtingen	Germany	89	Female	-20.2	9.7	Rib	9
Jechtingen	Germany	91	Male	-20.3	9.5	Tibia	9
Jechtingen	Germany	92	Juvenile	-20.5	9	Skull	9
Jechtingen	Germany	93	Juvenile	-19.8	12.6	Rib	9
Jechtingen	Germany	94A	-	-20.7	8.5	Rib	9
Jechtingen	Germany	94B	Male	-20.3	9.2	Radius	9
Jechtingen	Germany	96	Juvenile	-20.7	8	Clavicle	9
Jechtingen	Germany	97	Female	-20.7	9.1	Fibula	9
Jechtingen	Germany	99	Female	-20.3	9.6	Rib	9
Jechtingen	Germany	100	Juvenile	-20.6	8.3	Rib	9
Jechtingen	Germany	101	Juvenile	-20.2	8.4	Ulna	9
Karsdorf	Germany	115	Juvenile	-19.9	8.4	Rib	7
Karsdorf	Germany	301	Juvenile	-19.2	12.6	Rib	7
Karsdorf	Germany	529	Juvenile	-20.2	8.1	Rib	7
Karsdorf	Germany	305	Juvenile	-19.7	8.5	Rib	7
Karsdorf	Germany	95	Juvenile	-20.1	10.8	Rib	7
Karsdorf	Germany	509	Male	-20	9.1	Rib	7
Karsdorf	Germany	537	Male	-19.7	8.9	Rib	7
Karsdorf	Germany	611	Male	-20.2	9.1	Rib	7
Karsdorf	Germany	304	Male	-20.3	8.8	Rib	7
Karsdorf	Germany	300	Male	-20	8.7	Rib	7
Karsdorf	Germany	605	Male	-19.8	9	Rib	7
Karsdorf	Germany	170	Male	-20	9	Rib	7
Karsdorf	Germany	303	Male	-20.5	8.8	Rib	7
Karsdorf	Germany	122	Male	-19.9	9.3	Rib	7
Karsdorf	Germany	131	Male	-19.5	9.3	Rib	7
Karsdorf	Germany	430	Female	-20.1	8.4	Rib	7
Karsdorf	Germany	302	Female	-19.6	10.2	Rib	7
Karsdorf	Germany	169	Female	-20	9.1	Rib	7
Karsdorf	Germany	155	Female	-20.1	9.4	Rib	7
Karsdorf	Germany	158	Female	-20.2	6.3	Rib	7
Karsdorf	Germany	299	Female	-19.6	8.9	Rib	7
Kleinhadersdorf	Austria	G1A	Male	-19.5	9.3	Rib	2
Kleinhadersdorf	Austria	G1B	Female	-19.6	9.4	Rib	2
Kleinhadersdorf	Austria	G1C	Female	-20.1	9.2	Rib	2
Kleinhadersdorf	Austria	G7	Male	-19.6	9.5	Rib	2

Kleinhadersdorf	Austria	G8	Male	-19.9	8.7	Rib	2
Kleinhadersdorf	Austria	G9	Juvenile	-19.4	9.6	Long	2
Kleinhadersdorf	Austria	G12	Male	-19.7	9.6	Rib	2
Kleinhadersdorf	Austria	G14	Female	-20.2	8.9	Rib	2
Kleinhadersdorf	Austria	G15	Female	-19.5	9.8	Rib	2
Kleinhadersdorf	Austria	G16	Male	-19.6	8.8	Rib	2
Kleinhadersdorf	Austria	G17	Juvenile	-19.8	8.3	Rib	2
Kleinhadersdorf	Austria	G19	Male	-20.2	8.3	Rib	2
Kleinhadersdorf	Austria	3	Male	-19.4	10	Long	2
Kleinhadersdorf	Austria	5A	Female	-18.9	9.8	Rib	2
Kleinhadersdorf	Austria	7	Juvenile	-20	9.6	Long	2
Kleinhadersdorf	Austria	10	Male	-19.6	9.7	Long	2
Kleinhadersdorf	Austria	11	Juvenile	-19.8	9.1	Long	2
Kleinhadersdorf	Austria	17	Male	-19.8	9.8	Rib	2
Kleinhadersdorf	Austria	22	Juvenile	-19	11.9	Rib	2
Kleinhadersdorf	Austria	26	Juvenile	-19	12.4	Cranium	2
Kleinhadersdorf	Austria	29	Male	-20	10.2	Rib	2
Kleinhadersdorf	Austria	32	Female	-19.9	9.2	Rib	2
Kleinhadersdorf	Austria	40	Male	-19.9	10.4	Long	2
Kleinhadersdorf	Austria	43	Juvenile	-20.3	8.9	Long	2
Kleinhadersdorf	Austria	55	Female	-19.8	9.3	Rib	2
Kleinhadersdorf	Austria	56	Female	-20	9.8	Long	2
Kleinhadersdorf	Austria	57	Male	-20.1	9.9	Long	2
Kleinhadersdorf	Austria	59	Juvenile	-20.2	9.3	Long	2
Kleinhadersdorf	Austria	65	Female	-20	9.3	Long	2
Kleinhadersdorf	Austria	69	Male	-19.5	9.4	Long	2
Kleinhadersdorf	Austria	79	Male	-19.9	9.9	Long	2
Kleinhadersdorf	Austria	81A	Male	-19.6	10	Long	2
Kleinhadersdorf	Austria	90	Male	-19.7	10	Rib	2
Kleinhadersdorf	Austria	91A	Female	-19.6	10.3	Long	2
Nieder-Morlen	Germany	NM50	Juvenile	-19.4	11.8	Long	10
Nieder-Morlen	Germany	NM89A	Juvenile	-20.3	10.2	Long	10
Nieder-Morlen	Germany	NM427	Juvenile	-20.2	10	Long	10
Nieder-Morlen	Germany	NM932	Juvenile	-20.3	11.1	Long	10
Nieder-Morlen	Germany	NM1276B	Juvenile	-21	9.6	Long	10
Nieder-Morlen	Germany	NM1276C	Male	-20.8	10.1	Long	10
Nieder-Morlen	Germany	NM1320	Juvenile	-20.4	8.7	Long	10
Nieder-Morlen	Germany	NM1962	Male	-20.3	10.2	Long	10
Nieder-Morlen	Germany	NM2112	Female	-20.5	10	Long	10
Nieder-Morlen	Germany	NM2304	Juvenile	-19.5	12.8	Long	10
Nieder-Morlen	Germany	NM2399	Female	-20.1	9.7	Long	10
Nieder-Morlen	Germany	NM2506	Juvenile	-20.4	10.6	Long	10
Nitra Horné Krškany	Slovakia	1	Female	-20.2	9.7	Rib	3
Nitra Horné Krškany	Slovakia	3	Juvenile	-20	9.3	Rib	3
Nitra Horné Krškany	Slovakia	4	Male	-20	9.5	Rib	3
Nitra Horné Krškany	Slovakia	4A	Female	-19.9	10.3	Rib	3
Nitra Horné Krškany	Slovakia	4B	Juvenile	-19.8	10.4	Rib	3
Nitra Horné Krškany	Slovakia	5	Juvenile	-20	9.9	MC	3
Nitra Horné Krškany	Slovakia	6	Female	-20.1	10.5	Rib	3
Nitra Horné Krškany	Slovakia	8	Male	-19.9	11	Rib	3
Nitra Horné Krškany	Slovakia	9	Male	-20.4	9.7	Long	3
Nitra Horné Krškany	Slovakia	14	Female	-20.4	9.8	Rib	3

Nitra Horné Krškany	Slovakia	15	Female	-21	10.2	Rib	3
Nitra Horné Krškany	Slovakia	15b	Juvenile	-19.7	13	Rib	3
Nitra Horné Krškany	Slovakia	17	Male	-19.9	10.3	Rib	3
Nitra Horné Krškany	Slovakia	18	Female	-20.4	10.4	Rib	3
Nitra Horné Krškany	Slovakia	19	Male	-19.9	10.7	Rib	3
Nitra Horné Krškany	Slovakia	20	Female	-19.9	9.9	Rib	3
Nitra Horné Krškany	Slovakia	22	Female	-20.4	10	Rib	3
Nitra Horné Krškany	Slovakia	23	Juvenile	-20.3	9.6	Rib	3
Nitra Horné Krškany	Slovakia	24	Female	-20.3	10.5	Rib	3
Nitra Horné Krškany	Slovakia	25	Male	-20.2	10.5	Rib	3
Nitra Horné Krškany	Slovakia	26	Male	-20.1	10.5	Rib	3
Nitra Horné Krškany	Slovakia	27	Female	-20.4	10	Rib	3
Nitra Horné Krškany	Slovakia	28	Juvenile	-19.2	12.7	Cranium	3
Nitra Horné Krškany	Slovakia	29	Juvenile	-20.5	9.6	Rib	3
Nitra Horné Krškany	Slovakia	30	Juvenile	-19.9	10.1	Rib	3
Nitra Horné Krškany	Slovakia	31	Juvenile	-19.8	12.8	Rib	3
Nitra Horné Krškany	Slovakia	32	Female	-20.5	10.3	Rib	3
Nitra Horné Krškany	Slovakia	33	Female	-20.3	9.8	Rib	3
Nitra Horné Krškany	Slovakia	34	Male	-19.8	10.5	Rib	3
Nitra Horné Krškany	Slovakia	35	Female	-19.9	10.1	Rib	3
Nitra Horné Krškany	Slovakia	36	Female	-20.2	10.5	Rib	3
Nitra Horné Krškany	Slovakia	37	Female	-20.3	9.8	Rib	3
Nitra Horné Krškany	Slovakia	38	Juvenile	-20.6	10.1	Rib	3
Nitra Horné Krškany	Slovakia	39	Female	-19.8	10	Rib	3
Nitra Horné Krškany	Slovakia	41	Juvenile	-20.1	10.2	Rib	3
Nitra Horné Krškany	Slovakia	44	Female	-20.4	10.6	Rib	3
Nitra Horné Krškany	Slovakia	47	Juvenile	-19.4	12	Cranium	3
Nitra Horné Krškany	Slovakia	48	Female	-20.3	9.6	Rib	3
Nitra Horné Krškany	Slovakia	49	Juvenile	-20.3	9.5	Rib	3
Nitra Horné Krškany	Slovakia	50	Juvenile	-20.2	10.6	Cranium	3
Nitra Horné Krškany	Slovakia	52	Female	-20.2	9.8	Rib	3
Nitra Horné Krškany	Slovakia	53	Female	-20.1	10.6	Rib	3
Nitra Horné Krškany	Slovakia	54	Juvenile	-20.3	10.7	Rib	3
Nitra Horné Krškany	Slovakia	56	Male	-19.9	9.7	Rib	3
Nitra Horné Krškany	Slovakia	57	Female	-20.1	11.1	Rib	3
Nitra Horné Krškany	Slovakia	58	Male	-19.8	10.6	Rib	3
Nitra Horné Krškany	Slovakia	61	Female	-19.9	10.2	Rib	3
Nitra Horné Krškany	Slovakia	62	Male	-20.2	10.2	Rib	3
Nitra Horné Krškany	Slovakia	64	Female	-20.3	9.8	Rib	3
Nitra Horné Krškany	Slovakia	66	Female	-20.1	10	Long	3
Nitra Horné Krškany	Slovakia	67	Juvenile	-19.7	12.4	Rib	3
Nitra Horné Krškany	Slovakia	68	Juvenile	-20.4	9.8	Cranium	3
Nitra Horné Krškany	Slovakia	69	Male	-20	10.5	Rib	3
Nitra Horné Krškany	Slovakia	70	Female	-19.8	10.4	Rib	3
Nitra Horné Krškany	Slovakia	71	Juvenile	-20.2	10.6	Rib	3
Nitra Horné Krškany	Slovakia	72	Male	-20.4	10.7	Rib	3
Nitra Horné Krškany	Slovakia	74	Juvenile	-20.3	10.8	Rib	3
Nitra Horné Krškany	Slovakia	77	Male	-20.5	11	Long	3
Otzing	Germany	3	Juvenile	-20.4	8.1	Rib	11
Otzing	Germany	4	Female	-20.3	8.7	Rib	11
Otzing	Germany	5	Female	-19.7	8.9	Rib	11
Otzing	Germany	6	Female	-20.2	8.1	Rib	11

Otzing	Germany	10A	Female	-20.2	8.2	Rib	11
Otzing	Germany	14	Juvenile	-20.3	8.4	Rib	11
Otzing	Germany	17	Female	-20.1	8.8	Rib	11
Otzing	Germany	18	Juvenile	-20.4	7.6	Rib	11
Otzing	Germany	22A	Juvenile	-20.2	8.7	Rib	11
Otzing	Germany	26	Juvenile	-20.1	7.7	Rib	11
Otzing	Germany	27A	Juvenile	-20.1	8.3	Rib	11
Otzing	Germany	29A	Juvenile	-20.5	8.8	Rib	11
Otzing	Germany	29B	Juvenile	-20.7	8.1	Rib	11
Otzing	Germany	33	Juvenile	-20.3	8.5	Rib	11
Otzing	Germany	36	Juvenile	-20.6	7.7	Rib	11
Otzing	Germany	37	Juvenile	-20.3	8.4	Rib	11
Otzing	Germany	38	Juvenile	-20.5	7.9	Rib	11
Polgár-Ferenci-hát	Hungary	33/42	Female	-19.7	11	Rib	5
Polgár-Ferenci-hát	Hungary	34/82	Female	-20.2	10.6	Rib	5
Polgár-Ferenci-hát	Hungary	34/91	Juvenile	-19.7	10.3	Rib	5
Polgár-Ferenci-hát	Hungary	69/133	Female	-20.3	11	Rib	5
Polgár-Ferenci-hát	Hungary	103/208	Male	-19.6	9.8	Rib	5
Polgár-Ferenci-hát	Hungary	134/239	Juvenile	-20.3	9.5	Rib	5
Polgár-Ferenci-hát	Hungary	270/432	Male	-19.8	9.8	Rib	5
Polgár-Ferenci-hát	Hungary	281/444	Female	-20.1	10.6	Rib	5
Polgár-Ferenci-hát	Hungary	282/445	Male	-19.8	9.1	Rib	5
Polgár-Ferenci-hát	Hungary	285/450	Male	-20.1	10.3	Rib	5
Polgár-Ferenci-hát	Hungary	288/453	Juvenile	-18.7	13.6	Rib	5
Polgár-Ferenci-hát	Hungary	289/454	Juvenile	-19.7	10.7	Rib	5
Polgár-Ferenci-hát	Hungary	296/468	Female	-19.8	9.8	Rib	5
Polgár-Ferenci-hát	Hungary	311/486	Female	-19.9	10.5	Cranium	5
Polgár-Ferenci-hát	Hungary	314/489	Male	-20	10.9	Rib	5
Polgár-Ferenci-hát	Hungary	338/517	Juvenile	-20.2	9	Rib	5
Polgár-Ferenci-hát	Hungary	340/519	Female	-20.2	10.7	Rib	5
Polgár-Ferenci-hát	Hungary	341/520	Male	-20	10.1	Rib	5
Polgár-Ferenci-hát	Hungary	344/523	Female	-20.1	9.5	Cranium	5
Polgár-Ferenci-hát	Hungary	347/526	Juvenile	-20	10	Rib	5
Polgár-Ferenci-hát	Hungary	350/529	Juvenile	-19.6	10.5	Cranium	5
Polgár-Ferenci-hát	Hungary	353/532	Male	-20.2	9.8	Rib	5
Polgár-Ferenci-hát	Hungary	356/535	Male	-19.4	10.6	Rib	5
Polgár-Ferenci-hát	Hungary	368/562	Female	-20.4	9.7	Rib	5
Polgár-Ferenci-hát	Hungary	387/581	Male	-19.6	10.7	Rib	5
Polgár-Ferenci-hát	Hungary	448/646	Juvenile	-20	13.2	Long	5
Polgár-Ferenci-hát	Hungary	484/685	Juvenile	-20.1	10	Rib	5
Polgár-Ferenci-hát	Hungary	486/687	Male	-20.3	10.1	Rib	5
Polgár-Ferenci-hát	Hungary	644/944	Juvenile	-19.4	10.4	Rib	5
Polgár-Ferenci-hát	Hungary	697/1004	Male	-19.4	10.4	Long	5
Polgár-Ferenci-hát	Hungary	717/1024	Female	-20.7	9.9	Rib	5
Polgár-Ferenci-hát	Hungary	718/1025	Female	-19.8	9.9	Rib	5
Polgár-Ferenci-hát	Hungary	719/1026	Juvenile	-19.8	10.1	Rib	5
Polgár-Ferenci-hát	Hungary	721/1028	Male	-19.7	10.7	Rib	5
Polgár-Ferenci-hát	Hungary	764/1079	Male	-19.7	10	Rib	5
Polgár-Ferenci-hát	Hungary	773/1093	Female	-20.2	10.1	Rib	5
Polgár-Ferenci-hát	Hungary	786/1109	Juvenile	-19.5	14.2	Rib	5
Polgár-Ferenci-hát	Hungary	805/1138	Male	-20.5	10.6	Rib	5
Polgár-Ferenci-hát	Hungary	821/1168	Female	-20.1	10.8	Rib	5

Polgár-Ferenci-hát	Hungary	839/1198	Female	-20	11.4	Rib	5
Polgár-Ferenci-hát	Hungary	861/1222	Juvenile	-20	10.4	Rib	5
Polgár-Ferenci-hát	Hungary	867/1230	Male	-20.2	11.6	Rib	5
Polgár-Ferenci-hát	Hungary	871/1236	Juvenile	-19.8	12.6	Rib	5
Polgár-Ferenci-hát	Hungary	881/1252	Juvenile	-19.1	10.1	Long	5
Polgár-Ferenci-hát	Hungary	889/1260	Female	-20	10.5	Rib	5
Polgár-Ferenci-hát	Hungary	890/1261	Male	-19.6	9.8	Rib	5
Polgár-Ferenci-hát	Hungary	897/1268	Male	-19.7	10.2	Rib	5
Polgár-Ferenci-hát	Hungary	904/1311	Female	-19.3	9.6	Rib	5
Rutzing	Austria	4	Juvenile	-20.9	9.2	Cranium	2
Rutzing	Austria	5	Male	-20.7	9.7	Long	2
Rutzing	Austria	6	Juvenile	-20.7	9.1	Cranium	2
Rutzing	Austria	7	Juvenile	-20.3	11.6	Cranium	2
Rutzing	Austria	8	Male	-20.5	10	Rib	2
Rutzing	Austria	9	Female	-20.2	8.6	Long	2
Rutzing	Austria	10	Juvenile	-20.7	9.1	Long	2
Rutzing	Austria	13	Male	-20.2	9.8	Rib	2
Rutzing	Austria	14	Male	-21.1	9.6	Rib	2
Rutzing	Austria	15	Male	-20.5	9.4	Long	2
Rutzing	Austria	17	Female	-20.7	10.3	Long	2
Rutzing	Austria	24	Juvenile	-20.7	9.5	Rib	2
Rutzing	Austria	26	Juvenile	-20.8	8.5	Long	2
Rutzing	Austria	30	Female	-20.5	9.1	Long	2
Rutzing	Austria	31	Juvenile	-20.6	9.7	Rib	2
Rutzing	Austria	32	Male	-20.4	9.7	Rib	2
Schwetzingen	Germany	4	Male	-20.2	8.9	Rib	12
Schwetzingen	Germany	5	Male	-20.3	9.2	Rib	12
Schwetzingen	Germany	6	Male	-20.2	8.6	Rib	12
Schwetzingen	Germany	7	Male	-20.4	9.5	Phalanx	12
Schwetzingen	Germany	8	Male	-20.1	9.6	Rib	12
Schwetzingen	Germany	9	Female	-20.5	9.1	Rib	12
Schwetzingen	Germany	10	Male	-19.9	9	Rib	12
Schwetzingen	Germany	11	Female	-20.3	9	Rib	12
Schwetzingen	Germany	12	Juvenile	-20.3	8.2	Rib	12
Schwetzingen	Germany	14	Male	-20.2	9	Rib	12
Schwetzingen	Germany	16	Male	-20.1	9.9	Rib	12
Schwetzingen	Germany	18	Female	-20.2	9.1	Rib	12
Schwetzingen	Germany	19	Female	-20.2	8.7	Rib	12
Schwetzingen	Germany	21	Juvenile	-20.1	8	MT	12
Schwetzingen	Germany	25	Female	-20	8.1	Rib	12
Schwetzingen	Germany	26	Male	-20	9.1	Rib	12
Schwetzingen	Germany	28	Female	-20.2	8.6	Rib	12
Schwetzingen	Germany	30	Female	-20.4	8	Rib	12
Schwetzingen	Germany	31	Juvenile	-20.4	9.1	Rib	12
Schwetzingen	Germany	33	Juvenile	-20.4	9.1	Rib	12
Schwetzingen	Germany	35	Male	-20	9.1	Rib	12
Schwetzingen	Germany	37	Female	-20.1	8.7	Phalanx	12
Schwetzingen	Germany	38	Female	-20.1	8.3	Rib	12
Schwetzingen	Germany	39	Female	-20.3	9.1	Rib	12
Schwetzingen	Germany	41	Female	-20.2	8.6	Rib	12
Schwetzingen	Germany	42	Female	-20.4	8.7	Rib	12
Schwetzingen	Germany	43	Male	-19.8	9	Rib	12

Schwetzingen	Germany	45	Juvenile	-20.1	8.6	Rib	12
Schwetzingen	Germany	48	Female	-19.9	8.6	Rib	12
Schwetzingen	Germany	54	Female	-19.7	9.7	Rib	12
Schwetzingen	Germany	56	Male	-20.1	9.1	Rib	12
Schwetzingen	Germany	57	Female	-20.3	8.5	Rib	12
Schwetzingen	Germany	65	Female	-20.1	9.3	Rib	12
Schwetzingen	Germany	66	Male	-20.3	8.8	Rib	12
Schwetzingen	Germany	67	Female	-20.3	9.7	Rib	12
Schwetzingen	Germany	70	Male	-20.1	8.8	Rib	12
Schwetzingen	Germany	71	Female	-20.5	8.4	Rib	12
Schwetzingen	Germany	73	Male	-19.8	9.1	Rib	12
Schwetzingen	Germany	77	Male	-20.1	9.1	Rib	12
Schwetzingen	Germany	80	Male	-20.2	9.4	Rib	12
Schwetzingen	Germany	82	Male	-20.2	9.8	Rib	12
Schwetzingen	Germany	89	Female	-20.1	9.5	Rib	12
Schwetzingen	Germany	93	Male	-20.2	9.5	Rib	12
Schwetzingen	Germany	97	Male	-20.3	8.6	Rib	12
Schwetzingen	Germany	99	Female	-20.2	9.5	Rib	12
Schwetzingen	Germany	100	Female	-20.1	9.4	Rib	12
Schwetzingen	Germany	101	Female	-20.1	10.7	Rib	12
Schwetzingen	Germany	104	Male	-20.4	8.8	Rib	12
Schwetzingen	Germany	105	Female	-19.9	9.4	Rib	12
Schwetzingen	Germany	106	Male	-20.9	8.9	Rib	12
Schwetzingen	Germany	107	Male	-20.1	8.9	Rib	12
Schwetzingen	Germany	109	Female	-20.1	9.5	Rib	12
Schwetzingen	Germany	110	Male	-20.4	8.5	Rib	12
Schwetzingen	Germany	115	Juvenile	-20.4	9.2	Rib	12
Schwetzingen	Germany	119	Female	-20.1	8.4	Rib	12
Schwetzingen	Germany	120	Female	-20.3	8.8	Rib	12
Schwetzingen	Germany	121	Male	-20.3	8.9	Rib	12
Schwetzingen	Germany	122	Female	-20.3	9.3	Rib	12
Schwetzingen	Germany	123	Female	-20.3	8.3	Rib	12
Schwetzingen	Germany	125	Female	-20.5	8.8	Rib	12
Schwetzingen	Germany	126	Female	-20.1	8.1	Rib	12
Schwetzingen	Germany	128	Female	-20	9.9	Rib	12
Schwetzingen	Germany	129	Juvenile	-20.2	7.8	Rib	12
Schwetzingen	Germany	130	Male	-20	8.4	Rib	12
Schwetzingen	Germany	131	Female	-20.4	8.7	Rib	12
Schwetzingen	Germany	132	Male	-20.4	8.4	Rib	12
Schwetzingen	Germany	133	Male	-20	9.1	Rib	12
Schwetzingen	Germany	137	Female	-19.9	8.5	Rib	12
Schwetzingen	Germany	139	Male	-20	9.3	Rib	12
Schwetzingen	Germany	140	Female	-20	8.6	Rib	12
Schwetzingen	Germany	141	Female	-20.4	9.4	Rib	12
Schwetzingen	Germany	142	Male	-20.2	8.4	Rib	12
Schwetzingen	Germany	143	Male	-19.9	8.6	Rib	12
Schwetzingen	Germany	145	Female	-20.5	9.2	Rib	12
Schwetzingen	Germany	146	Female	-20.5	7.9	Rib	12
Schwetzingen	Germany	149	Female	-20.2	7.6	Rib	12
Schwetzingen	Germany	150	Female	-20.2	8.5	Rib	12
Schwetzingen	Germany	151	Female	-20.4	8.3	Rib	12
Schwetzingen	Germany	152	Male	-19.9	8.9	Rib	12

Schwetzingen	Germany	153	Female	-19.7	7.7	Rib	12
Schwetzingen	Germany	154	Male	-20.2	8.6	Rib	12
Schwetzingen	Germany	155	Juvenile	-20.3	7.8	Rib	12
Schwetzingen	Germany	157	Male	-19.9	8.4	Rib	12
Schwetzingen	Germany	163	Female	-20.2	9.7	Rib	12
Schwetzingen	Germany	169	Male	-20.1	8.9	Rib	12
Schwetzingen	Germany	170	Female	-20.1	7.9	Rib	12
Schwetzingen	Germany	171	Female	-20.1	9.5	Rib	12
Schwetzingen	Germany	174	Male	-19.6	8.3	Rib	12
Schwetzingen	Germany	176	Female	-20.1	10.1	Rib	12
Schwetzingen	Germany	177	Male	-20	9.2	Rib	12
Schwetzingen	Germany	183	Female	-20	8.7	Rib	12
Schwetzingen	Germany	184	Female	-19.9	9	Rib	12
Schwetzingen	Germany	185	Male	-20.3	9.8	Rib	12
Schwetzingen	Germany	188	Female	-20.2	8.6	Rib	12
Schwetzingen	Germany	189	Male	-20.1	8.9	Rib	12
Schwetzingen	Germany	190	Female	-20.2	9	Rib	12
Schwetzingen	Germany	194	Male	-19.7	8.3	Rib	12
Schwetzingen	Germany	199	Female	-20.4	9.2	Rib	12
Schwetzingen	Germany	200	Male	-19.6	9.2	Rib	12
Schwetzingen	Germany	201	Female	-20.2	8.7	Rib	12
Schwetzingen	Germany	218	Male	-20.3	8.8	Rib	12
Schwetzingen	Germany	219	Female	-20.1	8.8	Rib	12
Schwetzingen	Germany	220	Male	-19.7	8.4	Rib	12
Stuttgart-Mühlhausen	Germany	Avg	-	-20.5	9.7	Rib	9
Tesetice-Kyjovice	Czech Republic	11	Female	-19.6	9.5	Rib	3
Tesetice-Kyjovice	Czech Republic	15	Juvenile	-19.9	10.1	Rib	3
Tesetice-Kyjovice	Czech Republic	17	Juvenile	-19.5	8.8	Long	3
Tesetice-Kyjovice	Czech Republic	18	Female	-19.1	9.1	Rib	3
Tesetice-Kyjovice	Czech Republic	19	Male	-19.4	8.9	Rib	3
Tesetice-Kyjovice	Czech Republic	20	Male	-19.3	8.5	Rib	3
Tesetice-Kyjovice	Czech Republic	21	Male	-19.5	9	Rib	3
Vedrovice	Czech Republic	3(66)	Juvenile	-19.9	8.7	Rib	3
Vedrovice	Czech Republic	4(69)	Juvenile	-20.1	9.9	Rib	3
Vedrovice	Czech Republic	5(71)	Juvenile	-19.2	8.5	Rib	3
Vedrovice	Czech Republic	10(74)	Male	-19.9	9.6	Rib	3
Vedrovice	Czech Republic	13(75)	Female	-19.7	10.1	ns*	13
Vedrovice	Czech Republic	14(75)	Female	-19.3	10.1	ns*	13
Vedrovice	Czech Republic	15(75)	Male	-19.2	10	ns*	13
Vedrovice	Czech Republic	16(75)	Juvenile	-19.9	9.7	ns*	13
Vedrovice	Czech Republic	17(75)	Juvenile	-18.7	13.3	ns*	13
Vedrovice	Czech Republic	20/75	Juvenile	-19.7	8.4	Rib	3
Vedrovice	Czech Republic	21/75	Female	-19.6	9.2	Rib	3
Vedrovice	Czech Republic	22/75	Female	-19.8	9.5	ns*	13
Vedrovice	Czech Republic	23/75	Male	-20	10.6	ns*	13
Vedrovice	Czech Republic	24/75	Juvenile	-19.9	9.2	ns*	13
Vedrovice	Czech Republic	25/75	Male	-19.5	10	ns*	13
Vedrovice	Czech Republic	27/76	Female	-20.5	9.2	Rib	3
Vedrovice	Czech Republic	28/76	Juvenile	-19.9	10	ns*	13
Vedrovice	Czech Republic	29/76	Female	-20.5	9	Rib	3
Vedrovice	Czech Republic	30/76	Juvenile	-19.5	9.5	ns*	13
Vedrovice	Czech Republic	31/76	Female	-20	10.3	ns*	13

Vedrovice	Czech Republic	32/76	Juvenile	-19.9	8.5	Rib	3
Vedrovice	Czech Republic	36/76	Female	-19.7	8.8	Rib	3
Vedrovice	Czech Republic	37/76	Juvenile	-19.6	9.6	ns*	13
Vedrovice	Czech Republic	38/76	Female	-20.3	9.5	ns*	13
Vedrovice	Czech Republic	39/76	Juvenile	-19	10.5	ns*	13
Vedrovice	Czech Republic	40/76	Juvenile	-19.6	8.8	Long	3
Vedrovice	Czech Republic	42/77	Female	-19.5	9.8	ns*	13
Vedrovice	Czech Republic	43/77	Juvenile	-20	9.7	ns*	13
Vedrovice	Czech Republic	44/77	Juvenile	-19.9	9.5	ns*	13
Vedrovice	Czech Republic	46/77	Male	-19.8	9.1	Rib	3
Vedrovice	Czech Republic	48/77	Female	-19.8	10.3	ns*	13
Vedrovice	Czech Republic	50/77	Male	-19.8	9.5	ns*	13
Vedrovice	Czech Republic	51/77	Female	-20.7	9.5	ns*	13
Vedrovice	Czech Republic	54/78	Male	-19.6	10.1	ns*	13
Vedrovice	Czech Republic	56/78	Juvenile	-19.8	9.1	ns*	13
Vedrovice	Czech Republic	57/78	Male	-19.6	10.7	ns*	13
Vedrovice	Czech Republic	59/78	Male	-19.4	10.3	ns*	13
Vedrovice	Czech Republic	61/78	Female	-19.8	9.3	Rib	3
Vedrovice	Czech Republic	62/78	Female	-19.9	9.4	ns*	13
Vedrovice	Czech Republic	63/78	Male	-19.9	10	ns*	13
Vedrovice	Czech Republic	64/78	Female	-19.8	9.2	Rib	3
Vedrovice	Czech Republic	66/78	Male	-19.7	9.9	ns*	13
Vedrovice	Czech Republic	67/78	Female	-19.6	8.6	Rib	3
Vedrovice	Czech Republic	68/78	Female	-19.5	9.5	Rib	3
Vedrovice	Czech Republic	70/79	Female	-19.5	9.5	Rib	3
Vedrovice	Czech Republic	71/79	Male	-19.3	10.3	ns*	13
Vedrovice	Czech Republic	72/79	Female	-19.5	9.3	ns*	13
Vedrovice	Czech Republic	73/79	Male	-19.7	10.2	ns*	13
Vedrovice	Czech Republic	74/79	Female	-19.3	9.5	Rib	3
Vedrovice	Czech Republic	75/79	Female	-19.5	9.3	ns*	13
Vedrovice	Czech Republic	76/79	Male	-19.5	9.4	Rib	3
Vedrovice	Czech Republic	77/79	Male	-19.3	10	ns*	13
Vedrovice	Czech Republic	78/79	Juvenile	-19.5	9.1	Rib	3
Vedrovice	Czech Republic	79/79	Male	-19.6	10	ns*	13
Vedrovice	Czech Republic	80/79	Female	-19.6	9.2	Rib	3
Vedrovice	Czech Republic	81B/79	Juvenile	-20.1	10.4	ns*	13
Vedrovice	Czech Republic	82/79	Male	-19.1	10.6	ns*	13
Vedrovice	Czech Republic	83/80	Female	-19.5	9.2	Rib	3
Vedrovice	Czech Republic	84/80	Juvenile	-20.2	9.9	ns*	13
Vedrovice	Czech Republic	86/80	Female	-19.9	9.8	ns*	13
Vedrovice	Czech Republic	87/80	Female	-19.5	9.7	ns*	13
Vedrovice	Czech Republic	88/80	Male	-19.9	9.7	Rib	3
Vedrovice	Czech Republic	89/80	Female	-20	9.3	ns*	13
Vedrovice	Czech Republic	90/80	Female	-19.7	9.5	ns*	13
Vedrovice	Czech Republic	91/80	Female	-19.4	9.8	ns*	13
Vedrovice	Czech Republic	93A/80	Female	-19.8	10.2	ns*	13
Vedrovice	Czech Republic	94/80	Female	-19.7	9	Rib	3
Vedrovice	Czech Republic	95/80	Male	-19.7	9.7	ns*	13
Vedrovice	Czech Republic	96/80	Juvenile	-19.5	9.8	ns*	13
Vedrovice	Czech Republic	97/80	Female	-19.7	9.5	ns*	13
Vedrovice	Czech Republic	99/81	Male	-20.1	9.5	ns*	13
Vedrovice	Czech Republic	100/81	Female	-19.7	10	ns*	13

Vedrovice	Czech Republic	101/81	Female	-19.7	9.9	ns*	13
Vedrovice	Czech Republic	102/81	Female	-20	9.2	ns*	13
Vedrovice	Czech Republic	104/81	Female	-19.9	9.8	ns*	13
Vedrovice	Czech Republic	105/81	Juvenile	-19.7	9	ns*	13
Vedrovice	Czech Republic	106/82	Juvenile	-19.5	8.9	ns*	13
Vedrovice	Czech Republic	107/82	Female	-19.3	8.9	ns*	13
Vedrovice	Czech Republic	108/84	Male	-19.6	9.9	ns*	13
Vedrovice	Czech Republic	1(85)	Female	-20.5	9.7	Rib	3
Vedrovice	Czech Republic	2(85)	Male	-19.7	10.3	Rib	3
Vedrovice	Czech Republic	6(88)	Female	-20.2	9.6	Rib	3
Vedrovice	Czech Republic	7(88)	Female	-19.8	9.6	Rib	3
Vedrovice	Czech Republic	8(88)	Juvenile	-20.3	10.8	Rib	3
Vedrovice	Czech Republic	9(88)	Female	-20.2	9.7	Rib	3
Vedrovice	Czech Republic	10(89)	Female	-20.6	9.7	Rib	3
Vendenheim le Haut du Coteau	France	11A	Juvenile	-20.1	10	Rib	1
Vendenheim le Haut du Coteau	France	11B	Juvenile	-20	10.1	Rib	1
Vendenheim le Haut du Coteau	France	17	Male	-20.2	10.4	Long	1
Vendenheim le Haut du Coteau	France	21	Juvenile	-20.2	10.6	Rib	1
Vendenheim le Haut du Coteau	France	74	Male	-19.9	11	Rib	1
Vendenheim le Haut du Coteau	France	87	Juvenile	-20.1	11.1	Tibia	1
Vendenheim le Haut du Coteau	France	104	Male	-20.2	11	Rib	1
Vendenheim le Haut du Coteau	France	107	Juvenile	-20.3	11.4	Rib	1
Vendenheim le Haut du Coteau	France	131	Female	-20.1	8.9	Rib	1
Vendenheim le Haut du Coteau	France	133A	Juvenile	-20.2	10.2	Rib	1
Vendenheim le Haut du Coteau	France	133B	Juvenile	-20.2	10.3	Rib	1
Vendenheim le Haut du Coteau	France	139	Male	-19.8	11.1	Rib	1
Vendenheim le Haut du Coteau	France	141	Juvenile	-20.1	9.3	Rib	1
Vendenheim le Haut du Coteau	France	148	Male	-20.1	11.2	Rib	1
Vendenheim le Haut du Coteau	France	151	Male	-20.4	11	Rib	1
Vendenheim le Haut du Coteau	France	159	Juvenile	-20.3	9.9	Rib	1
Vendenheim le Haut du Coteau	France	161	Female	-20.3	11.1	Rib	1
Vendenheim le Haut du Coteau	France	172	Juvenile	-20.3	10.5	Rib	1
Vendenheim le Haut du Coteau	France	186	Male	-20.1	10.5	Rib	1
Vendenheim le Haut du Coteau	France	195	Juvenile	-20.6	10.7	Rib	1
Vendenheim le Haut du Coteau	France	199	Juvenile	-20.1	9.6	Long	1

Supplementary Table S7: Isotopic data for faunal remains plotted in Fig. 4

Site	Species	$\bar{x}$ $\delta^{13}C$	SD $\delta^{13}C$	$\bar{x}$ $\delta^{15}C$	SD $\delta^{15}C$	n	Source
Polgár-Ferenci-hát	Cattle	-19.8	0.54	8.4	1.04	13	5
Polgár-Ferenci-hát	Sheep/goat	-19.7	0.51	8.2	1.35	6	5
Polgár-Ferenci-hát	Pig	-20.2	0.5	9.4	0.74	6	5
Vedrovice	Cattle	-20.2	0.29	6.2	1.04	3	3
Vedrovice	Sheep/goat	-19.8	0.18	5.9	0.38	3	3
Vedrovice	Pig	-20.4	0.53	8.2	0.86	2	3
Nitra Horné Krškany	Cattle	-20.7	0.43	7.7	1.06	11	3
Nitra Horné Krškany	Sheep/goat	-20.1	0.3	7.3	1.64	6	3
Nitra Horné Krškany	Pig	-20.5	0.37	8.5	0.68	7	3
Heilbronn-Neckargartach	Cattle	-21.4	1.13	8.7	1	8	12
Heilbronn-Neckargartach	Sheep/goat	-20.3	0.55	8.5	0.54	4	12
Heilbronn-Neckargartach	Pig	-21.1	0.35	8.1	0.73	2	12

Supplementary Bibliography

1. Bickle, P. *et al.* in *The First Farmers of Central Europe: Diversity in LBK Lifeways* (eds. Bickle, P. & Whittle, A.) 159–204 (Oxbow Books, 2013).

2. Bickle, P. *et al.* in *The First Farmers of Central Europe: Diversity in LBK Lifeways* (eds. Bickle, P. & Whittle, A.) 291–342 (Oxbow Books, 2013).

3. Whittle, A. *et al.* in *The First Farmers of Central Europe: Diversity in LBK Lifeways* (eds. Bickle, P. & Whittle, A.) 49–100 (Oxbow Books, 2013).

4. Lillie, M. Vedrovice: demography and palaeopathology in an early farming population. *Anthropologie* **46**, 135-152 (2008).

5. Whittle, A. *et al.* in *The First Farmers of Central Europe: Diversity in LBK Lifeways* (eds. Bickle, P. & Whittle, A.) 101–158 (Oxbow Books, 2013).

6. Bickle, P. *et al.* Roots of diversity in a Linearbandkeramik community: isotope evidence at Aiterhofen (Bavaria, Germany). *Antiquity* **85,** 1243–1258 (2011).

7. Oelze, V. M. *et al.* Early Neolithic diet and animal husbandry: stable isotope evidence from three Linearbandkeramik (LBK) sites in Central Germany. *J. Archaeol. Sci.* **38,** 270–279 (2011).

8. Dürrwächter, C., Craig, O. E., Collins, M. J., Burger, J. & Alt, K. W. Beyond the grave: variability in Neolithic diets in Southern Germany? *J. Archaeol. Sci.* **33,** 39–48 (2006).

9. Mörseburg, A., Alt, K. W. & Knipper, C. Same old in Middle Neolithic diets? A stable isotope study of bone collagen from the burial community of Jechtingen, southwest Germany. *Journal of Anthropological Archaeology* **39,** 210–221 (2015).

10. Nehlich, O. *et al.* Mobility or migration: a case study from the Neolithic settlement of Nieder-Mörlen (Hessen, Germany). *J. Archaeol. Sci.* **36,** 1791–1799 (2009).

11. Hofmann, D. *et al.* in *The First Farmers of Central Europe: Diversity in LBK Lifeways* (eds. Bickle, P. & Whittle, A.) 205–250 (Oxbow Books, 2013).

12. Bentley, R. A. *et al.* in *The First Farmers of Central Europe: Diversity in LBK Lifeways* (eds. Bickle, P. & Whittle, A.) 251–290 (Oxbow Books, 2013).

13. Richards, M. P. *et al.* Isotopic analysis of humans and animals from Vedrovice. *Anthropologie* **46**, 185-194 (2008).
